# A bi-stage data-driven process-based model for sorghum breeding and yield prediction: coupling explainable artificial intelligence and crop modeling

**DOI:** 10.3389/fpls.2025.1617753

**Published:** 2026-01-08

**Authors:** Zheng Ni, Yanbin Chang, Joshua Kemp, Maria G. Salas-Fernandez, Lizhi Wang

**Affiliations:** 1 School of Industrial Engineering and Management, Oklahoma State University, Stillwater, OK, United States; 2 Department of Agronomy, Iowa State University, Ames, IA, United States; 3 Department of Bioengineering, George Mason University, Fairfax, VA, United States; 4 Department of Systems Engineering and Operations Research, George Mason University, Fairfax, VA, United States

**Keywords:** data-driven, process-based, crop modeling, explainable AI, neural network, GxE

## Abstract

With the global population explosion, the increasing demand in food supply pushes the development of advanced breeding methods. This study presents a bi-stage data-driven and process-based crop model to provide breeding recommendations based on Genotype x Environment (GxE) effects for sorghum, a vital cereal crop with various plant types, such as Grain (G), Forage (F), Dual Purpose (DP), and Photoperiod-Sensitive (PS). The model combines traditional process-based crop modeling with explainable data-driven methods, which increases the general interpretability and flexibility of the model. The model considers extensive environmental data, including seven years of hourly weather records and soil factors from three research farms in Iowa, together with management practices and parental information from 651 males and 131 females. Additionally, the model predicts the hourly dry weight of sorghum’s leaves, stems and grain, and predicts final yield based on management practices. The final combined Relative Root mean squared error reached 16% to 19% across several environmental conditions, which demonstrating the robust predictive capabilities. Besides, the model effectively identified elite hybrids in four distinct sorghum types, which also demonstrated its utility in reducing the need for extensive field trials. Additionally, our analysis of genotype by environment interactions revealed significant variability in performance, which indicates the precise breeding strategies customized for the environmental conditions are important and vital. This research highlights that our explainable hybrid model framework can greatly improve crop modeling and plant breeding, making agriculture more efficient and sustainable.

## 1 Introduction

Plant breeding is always a critical task in agriculture, focusing on improving crop varieties to enhance yield, resistance to pests and diseases, tolerance to environmental stresses, and nutritional quality (Ni et al., 2023; Khan et al., 2018; Hospital et al., 1992; Visscher et al., 1996; Bassi et al., 2024; Baranski et al., 2024). With the explosive global population and inevitable global warming, it becomes increasingly urgent to enhance crop productivity and resilience against environmental stressors (Dar and Laxmipathi Gowda, 2013; Serraj et al., 2019; Rockström et al., 2017; Bassi et al., 2024). Pursuing efficient and effective breeding methodologies is a vital and pressing task. However, this process is usually both time-consuming and costly. A typical breeding program requires multiple generations to implement breeding strategies, validate the crossings, and produce final commercial cultivars with stable and promising traits and yields (Ni et al., 2023; Ritland, 1984; Jarne and Charlesworth, 1993; Razanajatovo et al., 2016). A model capable of fast phenotypic prediction can help farmers forecast the future performance of the selected breeding lines under specific environmental settings. In this context, the development of advanced computational models holds tremendous promise in plant breeding (National Academies of Sciences, Engineering, and Medicine, 2019; Cobb et al., 2013).

Sorghum (*Sorghum bicolor* L. Moench) is one of the most important crops for enhanced agricultural productivity. With multiple plant types and uses, including Grain (G), Forage (F), Dual purpose (DP), and Photoperiod-Sensitive (PS), sorghum offers a massive genetic complexity for exploration (Panelo et al., 2024; Breitzman et al., 2019; Reddy et al., 2012). Each type of sorghum serves distinct agricultural purposes. Grain sorghum is primarily cultivated for grain production and is short to increase harvest index and to be amenable to mechanical harvest (Bean et al., 2013). The forage type is optimized for biomass accumulation and is widely used for livestock feed due to its high productivity and digestibility (Contreras-Govea et al., 2010). Dual-purpose type combines traits from both G and F types, balancing grain yield and biomass production, making it suitable for both grain harvesting and fodder (Bean et al., 2013; Rattunde et al., 2001). The PS type is the most interesting one among the four types, which is characterized by its exceptional biomass yield potential and height, often reaching more than 4 meters in non-tropical environments (Fernandez and Kemp, 2022; Panelo et al., 2024; Breitzman et al., 2019).

Our research is inspired by the biomass yield potential of the PS hybrids for the forage and bio-fuel industry and seeks to utilize advanced computational modeling techniques to identify potential PS hybrids with similarly outstanding traits (Panelo et al., 2024). However, unlike traditional breeding approaches that rely on genetic data from existing sorghum varieties (Hao et al., 2021; Xin et al., 2021; Jordan et al., 2011), our model offers a distinct advantage: we employ an explainable process-based model structure to generate synthetic genotypic patterns. By simulating genotypes instead of relying on observed genetic data, we can reduce the need for extensive genome sequencing and large-scale phenotype data collection and accelerate the breeding process.

A vast number of data-driven machine learning methods or process-based crop models have already been developed for yield prediction purposes. Machine learning methods, such as regression-based models, random forests, and neural networks, have been widely used in agriculture due to their great predictive capability. Process-based crop models, such as APSIM (Malone et al., 2007; Balboa et al., 2019; Keating et al., 2003) and DSSAT (Jones et al., 2003, 2011; Corbeels et al., 2016), simulate crop growth and development as complex interactions influenced by weather, soil, and management practices. These models utilize a human understanding of plant physiology and are readily interpretable through physiological mechanisms (Malone et al., 2007; Balboa et al., 2019). However, both sides have their limitations. While data-driven models typically treat genetics, environment, and management factors as plain numbers, which leads to a loss of interpretability and general applicability, process-based models suffer from poor predictive power when facing different varieties and environmental conditions (Chang et al., 2023). The training process for the process-based crop models is also time-consuming. Previous research combining data-driven methods and process-based crop models has already proven the advantage of an explainable data-driven model for yield prediction (Chang et al., 2023; Shahhosseini et al., 2021). In our other research, we have successfully constructed a hybrid crop model for sorghum yield prediction, which combines the data-driven and process-based techniques in one model framework (Chang et al., 2025). Here, we will extend the model framework to include the genetic component by combining explainable artificial intelligence (AI) and hybrid crop modeling for better predictive capability, generalizability, and interpretability.

**Figure 1:**
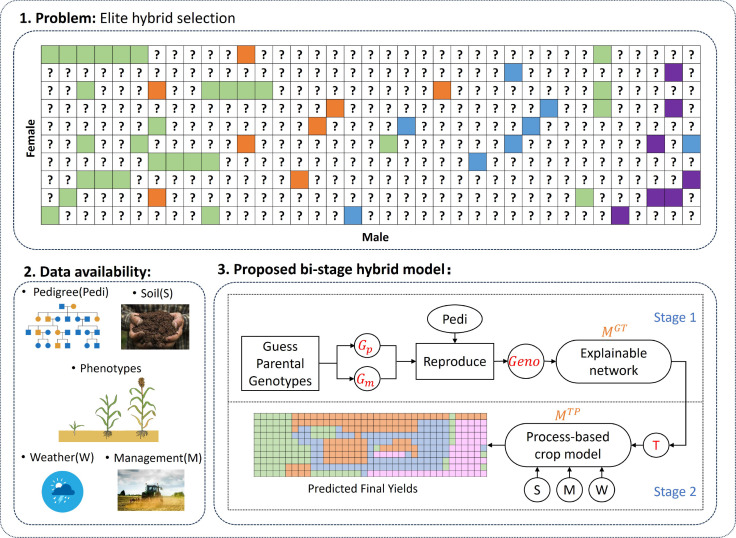
Brief introduction of the proposed bi-stage hybrid model. (*G_p_* and *G_m_* are the genotypes of the male and female. *M^GT^* and *M^TP^* are the two stages of our hybrid model, which defined in 2.3.).

Central to our approach is the development of a bi-stage hybrid model, as shown in **Figure 1**, a computational framework designed to simulate phenotypes and predict final yields across diverse sorghum hybrids. By integrating simulated genetic patterns, environmental factors, and management practices, our model could be a powerful tool for studying sorghum genetics and physiology and detecting high-performance individuals.

To achieve our research goal, we are confronted with the challenge of identifying and characterizing unseen sorghum hybrids based on limited data points available. To address this challenge, our methodology incorporates simulated genotypic patterns to identify the possible dominance and epistasis effect. Meanwhile, with a hybrid modeling approach that combines explainable data-driven modeling with process-based crop modeling, we want to provide a tool for more efficient decision-making. We use computational modeling and simulation to speed up genetic improvement in sorghum breeding. This helps reduce the time, and cost of traditional breeding methods. With our approach, we aim to better understand sorghum genetics and support a stronger, more sustainable future for agriculture.

In the following sections, we will first present the details of our proposed bi-stage hybrid crop model. Then, we will present the predicting capability and breeding application results.

## 2 Materials and methods

In this section, we will first present the data involved in this experiment and then present the detail of our proposed bi-stage hybrid model. The model is constructed for sorghum growth prediction and breeding recommendation. Our model will incorporate the knowledge of both genetics and plant physiology. We will also introduce a performance evaluation framework for F1 hybrids to compare our model with the traditional phenotypic selection strategy.

### 2.1 Data

#### 2.1.1 Environment data

Hourly weather data and soil data in three Iowa State University research farms in Iowa from 2015 to 2021 were collected from ISU Soil Moisture Network available through the Iowa Environmental Mesonet (IEM) (Herzmann et al., 2004). The abnormal data points are detected based on two sigma criteria in a continuous 20-sample window. Missing environmental data is a common challenge in meteorological and soil datasets, and various interpolation techniques have been proposed to address this issue (Cover and Hart, 1967; Wong et al., 2014; Liu and Gopalakrishnan, 2017; Yao et al., 2023). In this study, missing data was imputed using the k-Nearest-Neighbour (kNN) method (Cover and Hart, 1967), which has been widely used due to its non-parametric nature and ability to adapt to local data distributions. We utilized 13 weather parameters, air temperature, relative humidity, solar radiation, precipitation, wind speed, evapotranspiration, soil temperature (4 layers) and soil moisture (3 layers).

#### 2.1.2 Management data

Multiple management practices were utilized with different planting dates, harvest dates, and plant densities (plants/ft^2^). All the management data was collected at the farm-level.

#### 2.1.3 Phenotype data

Two data sets are utilized in our research. In the first set, plot-wise data were collected during harvest, including final yield, plant height and lodging scores. In our model, lodging score will be utilized as a penalization factor when generating the final yield. In the second set, data points were collected throughout the season by repeated measurements and single plant destructive measurements, including dry leaf weight, stem weight and plant height. Additionally, plot-wise final yield and lodging scores were recorded at the end of the season.

During the preprocessing, we removed all missing values and hybrids with the outlier values. For replicate experiments conducted under the same genotype, environmental settings, and management practices, records were aggregated by taking the mean value to represent a single unique sorghum sample. After the preprocessing, a total of 5149 series of sorghum samples from four distinct plant types, Grain, Dual-Purpose, Forage, and Photoperiod-Sensitive, are collected from 2015 to 2021. Among them, 200 samples with destructive measurements provide detailed daily phenotype data during the growth process, while the other 4949 samples provide the plot-wise phenotype data only. 1474 distinct hybrids obtained by crossing 651 males and 131 females are included in the final data. In this research, we will try to detect the elite hybrid from all 651×131 potential hybrids based on the provided data points.

### 2.2 Bi-stage hybrid model for sorghum growth

**Figure 2:**
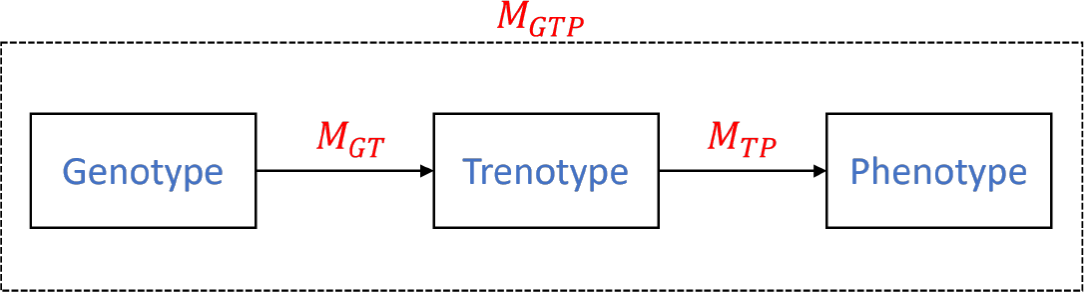
Main elements in the proposed bi-stage crop model.

In this section, we will introduce our proposed bi-stage hybrid model. The model considers Genotype × Environment × Management (G×E×M) effects and follows a general structure from Genotype → Trenotype → Phenotype (**Figure 2**). Here we define trenotype (T) as a translated genotype representation, a set of intermediate parameters derived from genotype and optimized through the model, which serves as the bridge between genomic data and phenotypic traits. Then, our model can be divided into two distinct stages, *M_GT_* and *M_TP_*.

#### 2.2.1 First stage (*M_GT)_*: pathway from gene to trenotype

**Figure 3:**
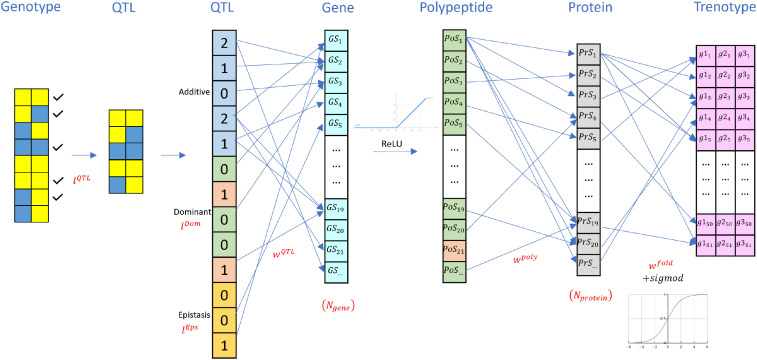
Detailed structure of the first layer of the simulation model (*M_GT_*).

Here, we start by introducing the first stage *M_GT_*, which follows the pathway from genotype to trenotype. This stage reflects the general biological path: genome - QTL - gene - polypeptide - protein - trenotype, focusing on the protein-coding gene products that contribute to the phenotype. However, it is important to note that some genes do not follow this pathway, as their transcripts function directly as regulators or signaling molecules. In our model, we preserved these dynamics by maintaining the linear relationships within the layers. While this model simplifies these aspects, it primarily aims to simulate the genotype-to-trenotype process in diploid plants, including the potential for F1 hybrid phenotype simulation. The general structure of this model is shown in **Figure 3**. Five main layers of this model are demonstrated as follows:

##### 2.2.1.1 SNP – QTL mapping

We start from the genotype data *G* in the first layer of the *M_GT_* model, which is a binary matrix with dimension *N* \× *N__g_\×* 2. * A* subset of loci are selected as quantitative trait loci (QTLs) through a binary selector vector *l^QTL^* ∈ {0,1}*^N_G^*, which activates or deactivates loci. By default, about 60% of all loci are selected as candidate QTLs (Shabalin, 2012). Among these selected loci, a further binary vector *l^Dom^* ∈{0,1}*^N_G^* specifies which loci can exhibit dominance effects, with approximately 30% of the selected QTLs initialized as dominant. In addition, pairwise epistasis is controlled by a sparse binary matrix $l^{Eps}\in {\left\{0,1\right\}}^{N_{G}\times N_{G}}$, where $l_{jk}^{Eps}=1$ indicates that loci *j* and *k* may jointly contribute to phenotype expression. All three selector parameters (*l^QTL^,l^Dom^,l^Eps^*) are randomly initialized and then lightly optimized during training, allowing both locations and interaction patterns to adapt to the population and environmental context rather than being fixed. This design ensures flexibility while maintaining biological plausibility. Consequently, the model accounts for additive, dominant, and a small number of epistasis effects (Voigt et al., 1966; Narain et al., 2007; Ishimori et al., 2020; Xu, 2003). The additive dosage, dominance indicator, and epistasis features are defined as (Equations 1–3):

(1) $$a_{j}=l_{j}^{QTL}\cdot \left(G_{n,j,1}+G_{n,j,2}\right),\;$$

(2) $$d_{j}=l_{j}^{QTL}\cdot l_{j}^{Dom}\cdot I\left(G_{n,j,1}\neq G_{n,j,2}\right),\;$$

(3) $$e_{jk}=l_{j}^{QTL}\cdot l_{k}^{QTL}\cdot l_{jk}^{Eps}\cdot I\left(a_{j}\geq 1\right)\cdot I\left(a_{k}\geq 1\right).$$

All additive, dominant, and epistasis features are concatenated into a QTL feature vector as Equation 4:

(4) $$x^{QTL}=a⊕d⊕e.$$

##### 2.2.1.2 QTL – gene mapping

Given the QTL feature vector *x^QTL^*, the contribution of QTLs to each protein-coding gene is modeled through a trainable weight vector $w_{i}^{QTL}$. This weight vector captures how the selected additive, dominant, and epistatic features influence gene expression. The gene score (*GS*) for gene *i* is then calculated as shown in Equation 5:

(5) $$GS_{i}={\left(w_{i}^{QTL}\right)}^{⊤}x^{QTL}.$$

##### 2.2.1.3 Gene - polypeptide mapping

For the second layer, we utilize the “One gene, one polypeptide” hypothesis as the basic assumption (Allaby, 2012; Fang et al., 2022). All the genes will only map to one potential polypeptide in our model. The amount of the polypeptide will be determined by the *GS* we obtained in the first layer. While the *GS* is lower than 0, we assume that the accumulated amount of polypeptide is too small for functional consideration. A ReLU (Equation 6) function is utilized here for math calculation. The outcome of this layer is defined as the polypeptide score (*PoS*):

(6) $$PoS_{i}=\text{ReLU}\left(GS_{i}\right).$$

##### 2.2.1.4 Polypeptide - protein mapping

For the third layer, we describe the generation of protein based on existing polypeptides. While one polypeptide can contribute to multiple proteins, some will only participate in the generation of one specific protein (Fang et al., 2022). Here, *w^poly^* is defined as the mapping from *PoS* to the Protein scores (*PrS*) as shown in Equation 7:

(7) $$PrS_{k}=\sum_{i}w_{ik}^{poly}\cdot PoS_{i}.$$

##### 2.2.1.5 Protein folding and interaction

For the fourth layer, proteins are folded and bring functionality to the sorghum growth (Fang et al., 2022; Burjoski and Reddy, 2021). *w^fold^* is defined as the folding and interaction effects for the proteins. To ensure the final scores are explainable and reasonable for the second stage, the descriptive crop model part, a sigmoid function is applied to scale all the effects to [0, 1]. Then, reweighing based on the bounds of trenotypes as applied as shown in Equation 8:

(8) $$T_{m}=\sigma \left(\sum_{k}w_{km}^{fold}\cdot PrS_{k}\right).$$

The whole first stage model, $M_{GT}$, can be formulated as function Equations 9, 10:

(9) $$T=f^{GT}\left(g,l^{QTL},l^{Dom},l^{Eps},w^{QTL},w^{poly},w^{fold}\right)$$

(10) $$=f^{GT}\left(g,l,w\right)$$

where

*T* is the trenotype of the sorghum individual,

*g* is the genotype of the sorghum individual,

*l^QTL^* is the locations of the QTLs in the genotype,

*w^QTL^* is the map from QTL to polypeptide score,

*l^Dom^* is the locations of the dominant effects in the genotype,

*l^Eps^* is the locations of the epistasis effects in the genotype,

*w^poly^* is the map from polypeptide score to protein score,

*w^fold^* is the map from protein score to trenotype,

*l* is the collection of all the location parameters,

*w* is the collection of all the weight parameters,

*f^GT^*(·) is the function defined above and shown in **Figure 3**.

#### 2.2.2 Second stage (*M_TP_*): pathway from trenotype to phenotype

In this section, we introduce the second stage *M_TP_*, which links the trenotype (*T*) to the phenotype. We adopt a previously developed data-driven crop model as the prototype (Chang et al., 2025), which captures nutrient and water flows in sorghum through a set of physiologically meaningful parameters. By coupling the first-stage output (*T*) with this descriptive crop model, we can directly evaluate individual-level phenotypes from their genetic background under specific soil, weather, and management conditions.

**Figure 4:**
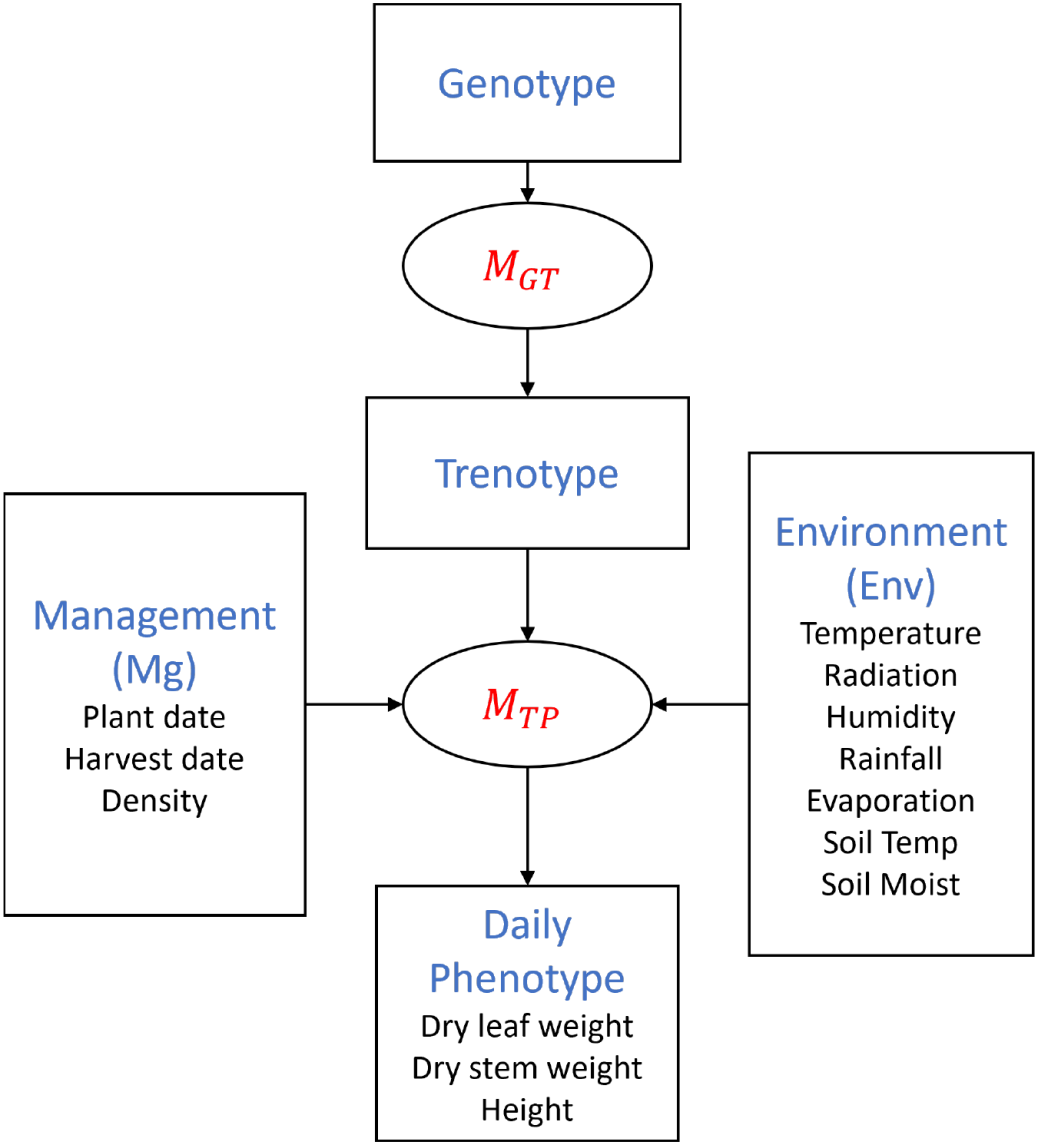
Elements of the second layer of the simulation model (*M_TP_*).

As shown in **Figure 4**, the second-stage model, *M_TP_*, uses hourly soil (*S*), weather (*W*), management data (*M*), and trenotypes (*T*) as the inputs. Meanwhile, it will generate the daily phenotypes, like dry leaf, root, and stem weight, wet leaf, root, and stem weight, and height, based on the daily growing degree unit (GDU) accumulation (Chang et al., 2025). The GDU is determined by hourly temperature, which is affected by weather fluctuations Chang et al. (2025). The detailed description and evaluation of the model can be found in Chang et al. (2025). In this research, we will focus on the hourly dry whole plant biomass (*b_t_*) and plant height (*h_t_*) within the first 180 days after planting, and the final plot yield (*y*).

The second stage model can be formulated as the function below As the function Equation 11:

(11) $$({\left\{b_{t}\right\}}_{t=1}^{4320},{\left\{h_{t}\right\}}_{t=1}^{4320},y)=P=f^{TP}\left(T,S,M,W\right)$$

where

*f^TP^*(·) is the function defined in Chang et al. (2025),

*P* is the phenotypes of the sorghum individual in daily units,

*b_t_* is the whole plant dry biomass at hour *t* after planting.

*h_t_* is the height of the sorghum at hour *t* after planting.

*y* is the final yield.

*T* is the trenotypes of the sorghum individual,

*S* is the sequence of hourly soil data,

*M* is the plot-wise management data, including plant dates and harvest dates,

*W* is the sequence of hourly weather data.

#### 2.2.3 Model calibration

By coupling the first-stage model *M^GT^* and second stage-model *M^TP^*, we can formulate the whole bi-stage framework *M^GTP^* as Equations 12–14:

(12) $$P=f^{TP}\left(T,S,M,W\right)$$

(13) $$\;=f^{TP}\left(f^{GT}\left(g,l,w\right),S,M,W\right)$$

(14) $$\;=f^{GTP}\left(g,l,w,S,M,W\right)$$

The coupled bi-stage hybrid model *M^GTP^* allows us to easily predict the growth curves of sorghum individuals based on simulated genotypes. To ensure adherence to natural patterns, calibration of parameters *g*, *l*, and *w* is conducted to ascertain reasonable trenotype *t* parameters, guided by historical data as shown in Equation 15:

(15) $$\hat{P}=f^{GTP}\left(\hat{g},\hat{l},\hat{w},S,M,W\right)$$

Evaluation of predicted phenotypes $\hat{P}$ is conducted through the utilization of a combined relative root-mean-square error (RRMSE) as the loss function as a loss function shown in Equation 16:

(16) $$L\left(P,\hat{P}\right)=\frac{1}{3}\text{RRMSE}\left(b_{t},{\hat{b}}_{t}\right)+\frac{1}{3}\text{RRMSE}\left(h_{t},{\hat{h}}_{t}\right)+\frac{1}{3}\text{RRMSE}\left(y,\hat{y}\right)$$

where

(17) $$\text{RRMSE}\left(x,\hat{x}\right)=\frac{\sqrt{\sum_{i=1}^{n}{\left(x_{i}-{\hat{x}}_{i}\right)}^{2}/n}}{\sum_{i=1}^{n}x_{i}/n}.$$

### 2.3 F1 Hybrid performance evaluation

#### 2.3.1 Traditional phenotypic selection method

To evaluate the performance of our model, a benchmark method is constructed based on the traditional Phenotypic Selection (TS) strategy. For the TS method, male and female scores are initially generated based on the mean value of their existing progeny’s phenotypes. If no progeny is observed, a score of 0 is assigned.

(18) $$FS_{i}=\frac{1}{n_{i}}\sum_{j}P_{ij},\;i=1,\;2,\;\ldots ,\;651$$

(19) $$MS_{j}=\frac{1}{n_{j}}\sum_{i}P_{ij},\;j=1,\;2,\ldots ,\;131$$

(20) $$TS_{ij}=FS_{i}+MS_{j},\;i=1,\;2,\ldots ,\;651,\;j=1,\;2,\ldots ,\;131$$

Here, *FS_i_* and *MS_j_* are the phenotypic scores for the male line *i* and female line *j* respectively. Equations 18 and 19 are utilized to generate the parents score based on the mean value of their progenies’ phenotypes (*P_ij_*). Here, *n_i_* and *n_j_* are the number of the existed hybrids for male *i* and female *j*. Equation 20 requires that the hybrid scores are the summation of their parents’ scores. Then, the cross score is calculated as the average of the parents’ scores. The pairs with the highest scores are selected for future breeding. This method can be formalized as the following integer linear programming (ILP) problem:

(21) $$\underset{I_{ij}}{\max}\;\sum_{i,j}TS_{ij}\cdot I_{ij}$$

(22) $$\text{s}.\text{t}.\;\sum_{i,j}I_{ij}=n$$

(23) $$I_{ij}\in \left\{0,1\right\},\;\;i=1,\;2,\ldots ,\;651,j=1,\;2,\ldots ,\;131$$

In this ILP, decision variable *I_i,j_* indicates whether (*I_i,j_* = 1) or not (*I_i,j_* = 0) hybrid generated by male line *i* and female line *j* is selected for breeding, as shown in Equation 23. *TS_ij_* is the phenotypic scores for the hybrid generated by male line *i* and female line *j*. The object function (Equation 21) maximizes the total performance of the selected hybrids. Constraint (Equation 22) limits the number of selected hybrids to a pre-set constant *n*.

#### 2.3.2 F1 hybrid prediction based on the proposed bi-stage model

This section introduces an evaluation framework to use our proposed model to evaluate the performance of the potential F1 hybrid under different environmental conditions. Our preprocessed dataset comprises 651 male lines and 131 female lines, representing a potential of 93,765 unique F1 hybrids. However, empirical data is only available for 1,475 of these hybrids, collected over a span of seven years. The primary challenge is to explore the potential of the remaining hybrids. The phenotypic selection method relies on observable traits from the limited data of 1,475 hybrids, a process that is considerably resource-intensive. In contrast, the proposed hybrid model predicts the performance of these hybrids by integrating genetic and environmental data, which can potentially accelerate the breeding process by reducing dependence on field trials.

Within this framework, we make the assumption that all parents are homozygous. Most of the samples share a male or female with other hybrids, which provides a linkage between their genotype coding. Leveraging the parental information including the male and female ID, we generate F1 offspring based on the genotypes of the parents.

For parental generation, the genotype will be considered as unknown parameters and jointly calibrated with parameters *l* and *w* to align with historical data. For hybrid generation, genotypes are generated based on the Parental information as shown in Equation 24:

(24) $$\hat{g}=f^{\text{Reproduce}}\left(g^{F},g^{M},ParentID\right)$$

where

*f*^Reproduce^(·) is the function that the reproducing process from parents’ information to the F1 population based on parental information.

*g^F^* is the genotype of the males.

*g^M^* is the genotype of the females.

*ParentID* refers to female and male ID.

Then, we will get the predicted phenotypes $\hat{P}$ through the GTP model as shown in Equations 25, 26:

(25) $$\hat{t}=f^{GT}\left(\hat{g},l,w\right)$$

(26) $$\hat{P}=f^{TP}\left(\hat{t},S,M,W\right)$$

Following this, calibration of parameters *l* and *w* is required to train the model. The trained models will be utilized for yield prediction for the selected hybrids. All the potential hybrids’ predicted phenotypes will be fed to the TS framework as we discussed in the section 2.4.1. The predicted phenotype $\hat{P}$ will replace the *P* in Equations 18 and 19. Then, optimal hybrids with the best *PS* scores will be recommended for future breeding.

## 3 Results

### 3.1 Predictive capability of the bi-stage hybrid model

To evaluate our proposed bi-stage hybrid model, we trained the model using the data detailed in the section 2.2. The data is split into 60% training set and 40% testing set. Here, the bi-stage hybrid model considered a total of 2000 alleles. Initially, the model underwent pre-training based on four distinct sorghum types individually to expedite the process, followed by retraining on the entire training population. The final training combined relative root mean square error (cRRMSE) stood at 12.6% and testing at 18.1%.

**Figure 5:**
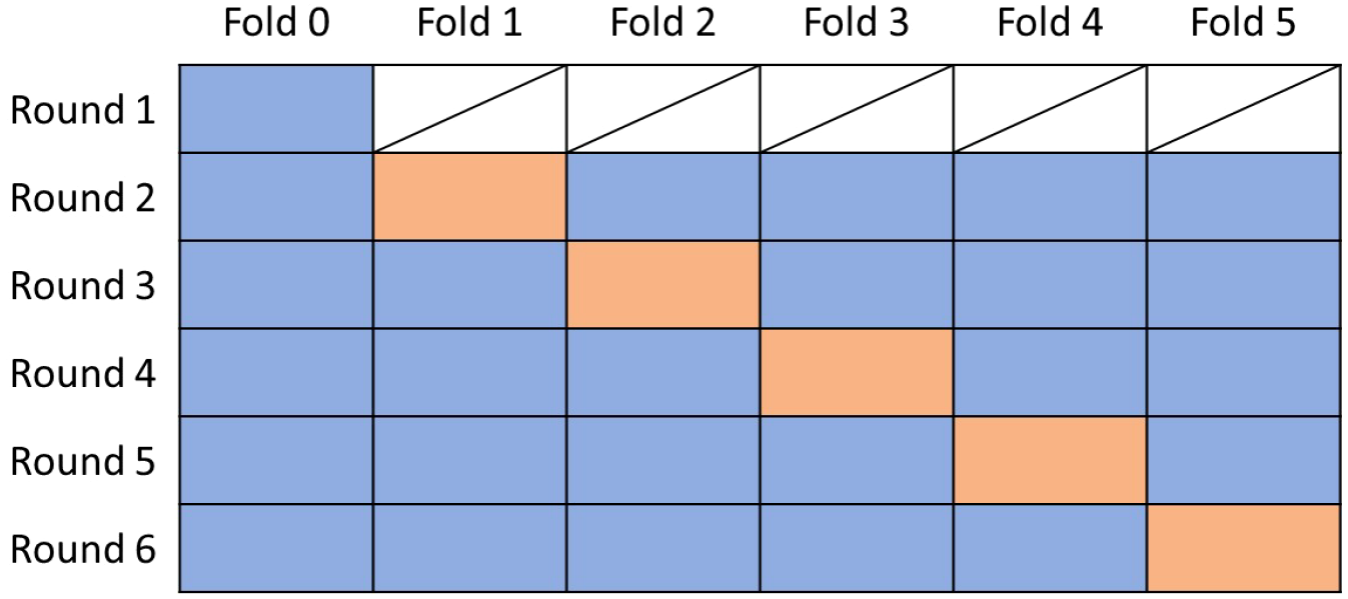
Arrangement for the cross validation. Blue fold is used for training and orange is used for testing.

**Figure 6:**
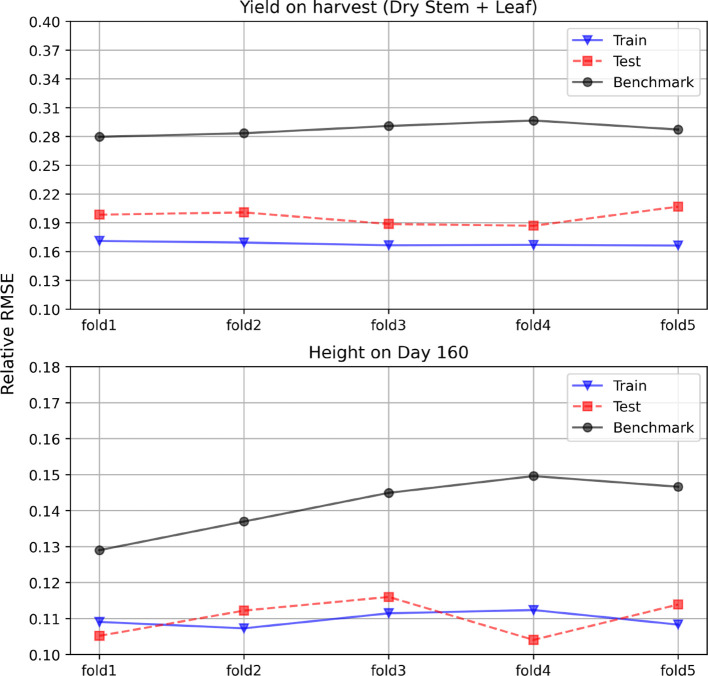
Model performance in 5-fold cross-validation: Comparison of relative RMSE for Dry Stem + Leaf Yield at harvest and plant height on day 160. Benchmark is the test result for traditional phenotypic selection.

To verify the robustness of our proposed method, we also performed cross validation to compare our model with the traditional phenotypic selection. The whole data is split into 6 folds. We used the fold 0 to pretrain the proposed model. The structure of the cross validation is shown as **Figure 5**. **Figure 6** shows the results for the cross validation. From the plot, we can see that the proposed hybrid model outperforms the benchmark on all folds, which achieved a stable 19% test RRMSE on the final yield and around 11% on the plant height prediction.

**Figure 7:**
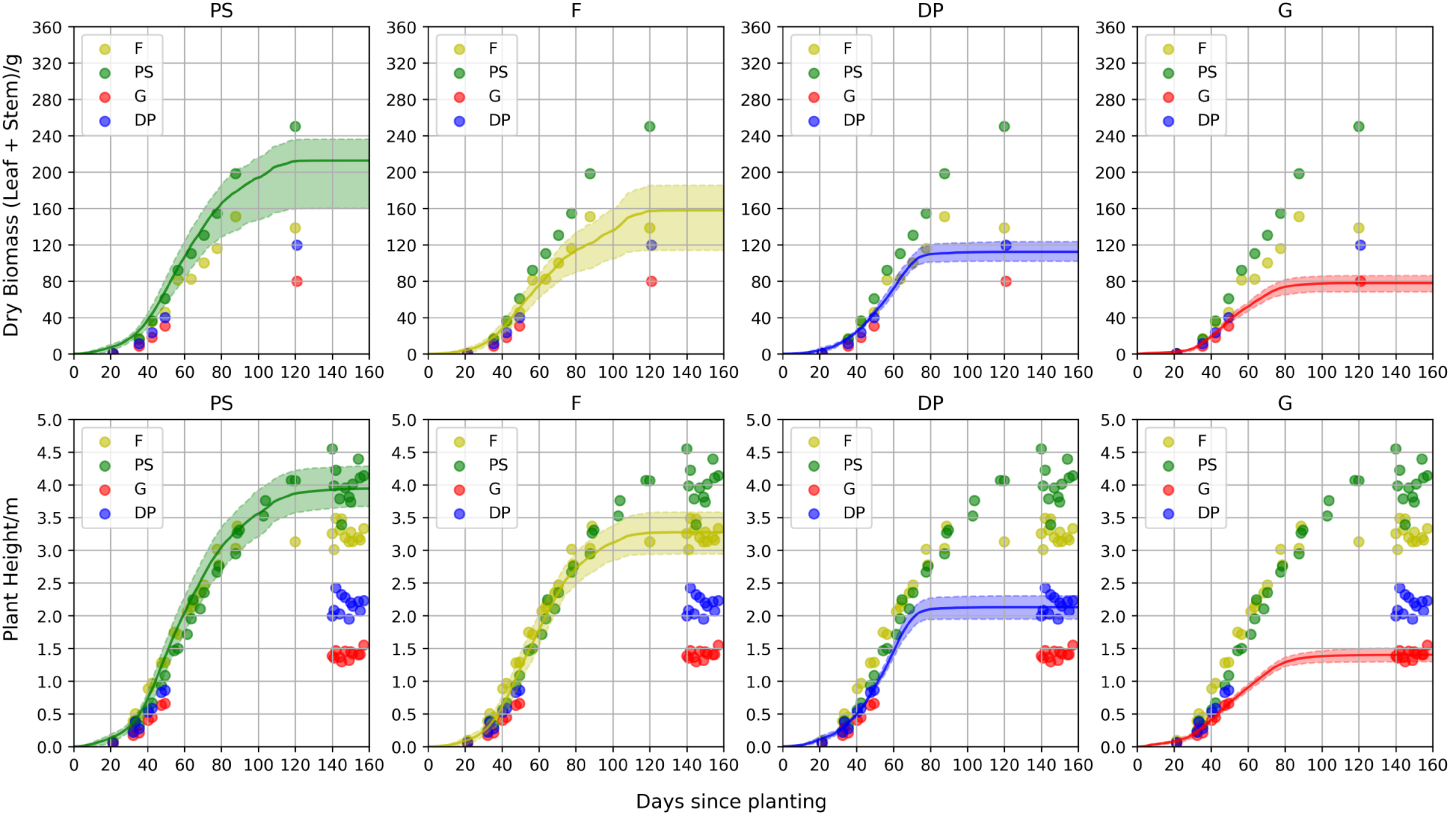
Observed biomass and height records and the predicted growth curves of four different plant types. The points represent the mean values of observed data collected on specific days. The curve shows the mean predicted values from the model, corresponding to biomass or height on the same days, while the shaded area represents the first and third quantiles.

**Figure 7** illustrates the predicted biomass (dry stem and leaf) and height curves for four different plant types together with the true biomass points. The points represent the mean values of the observed data collected on provided days. The curve shows the mean predicted values from the model, corresponding to biomass or height on the same days, while the shaded area represents the first and third quantiles. The growth of the sorghum reached a plateau for all sorghum types. While PS reached the plateau in the height curve after 120 days, the Forage type hits an early one after around 100. Our model clearly captured this pattern. Meanwhile, for the grain type and dual-purpose type, they naturally begin to produce grains after around 80 days, which leads to an earlier height growth plateau. Our model also gave a reasonable growth curve even with no process data provided. Evidently, the predicted sorghum individuals share a similar and reasonable growth pattern to their real-world counterparts, confirming the reliability of our bi-stage hybrid model.

### 3.2 Hybrid selection in different sorghum types

After illustrating the predictive capability of our model, we utilize the model to detect the elite hybrid among all given potential pairs. For different plant type, we choose different criteria.

- PS and F: Focusing on biomass weight (leaf and stem) on day 140 aligns with their utility in producing substantial vegetative growth, which is valuable for forage and bio-energy applications.
- G: Focusing on grain weight on day 140 ensures that selection prioritizes high-yield grain production, crucial for food and possibly feed purposes.
- DP: Evaluating the whole weight (leaf, stem, and grain) by Day 140 is a comprehensive approach that supports their dual-use for both grain and forage production, maximizing overall biomass and yield.

**Figure 8:**
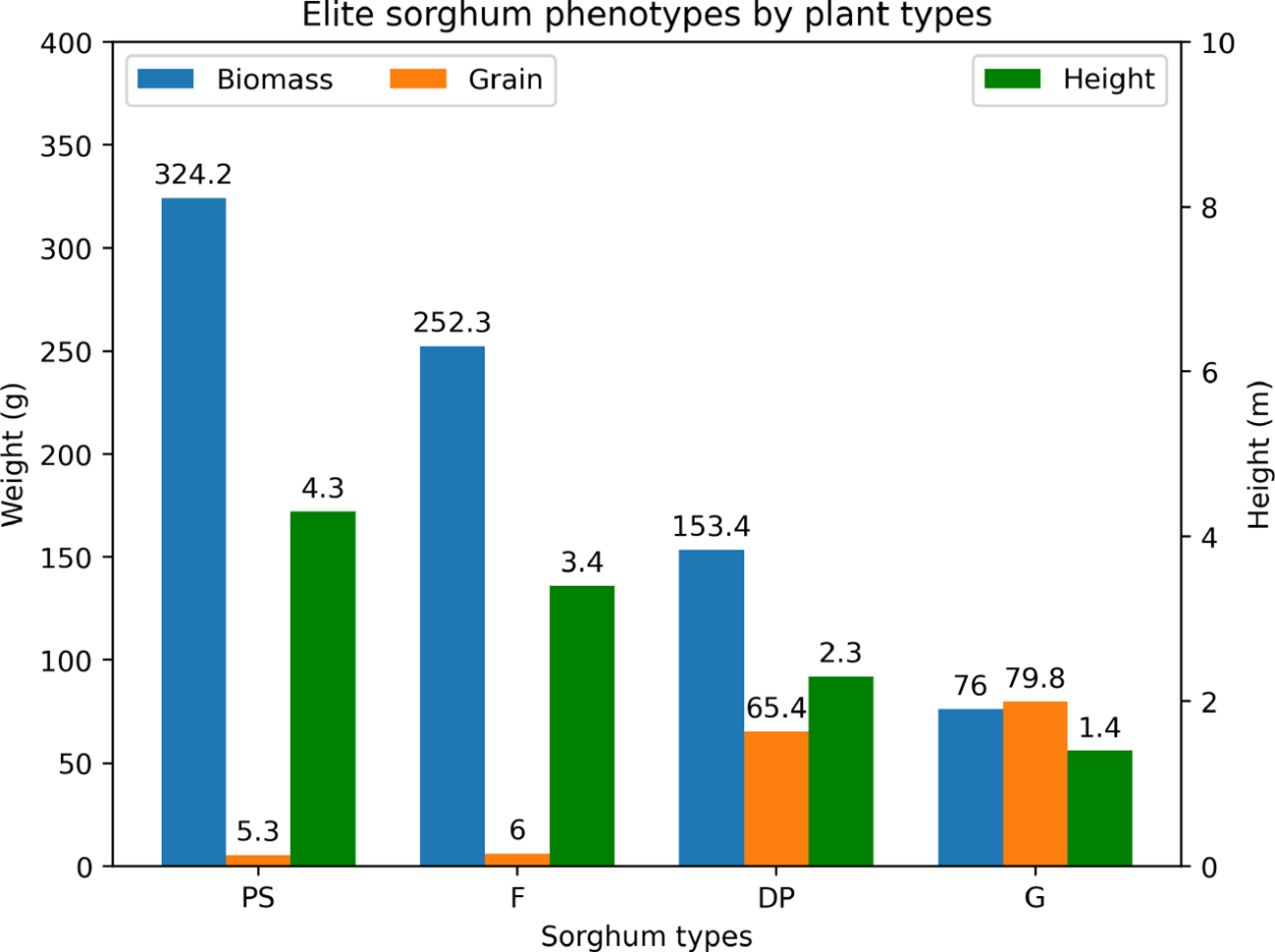
The predicted phenotypes of the selected elite hybrid per plant among different types.

Based on the different criterion, we picked the elite sorghum individuals. **Figure 8** compares the selected elite sorghum phenotypes in terms of biomass, grain weight, and height by day 140 across four plant types. The chart provides a clear visual summary of the strengths and trade-offs associated with each phenotype. While PS and F produce more substantial biomass, G and DP generate more grains as we expected.

**Figure 9:**
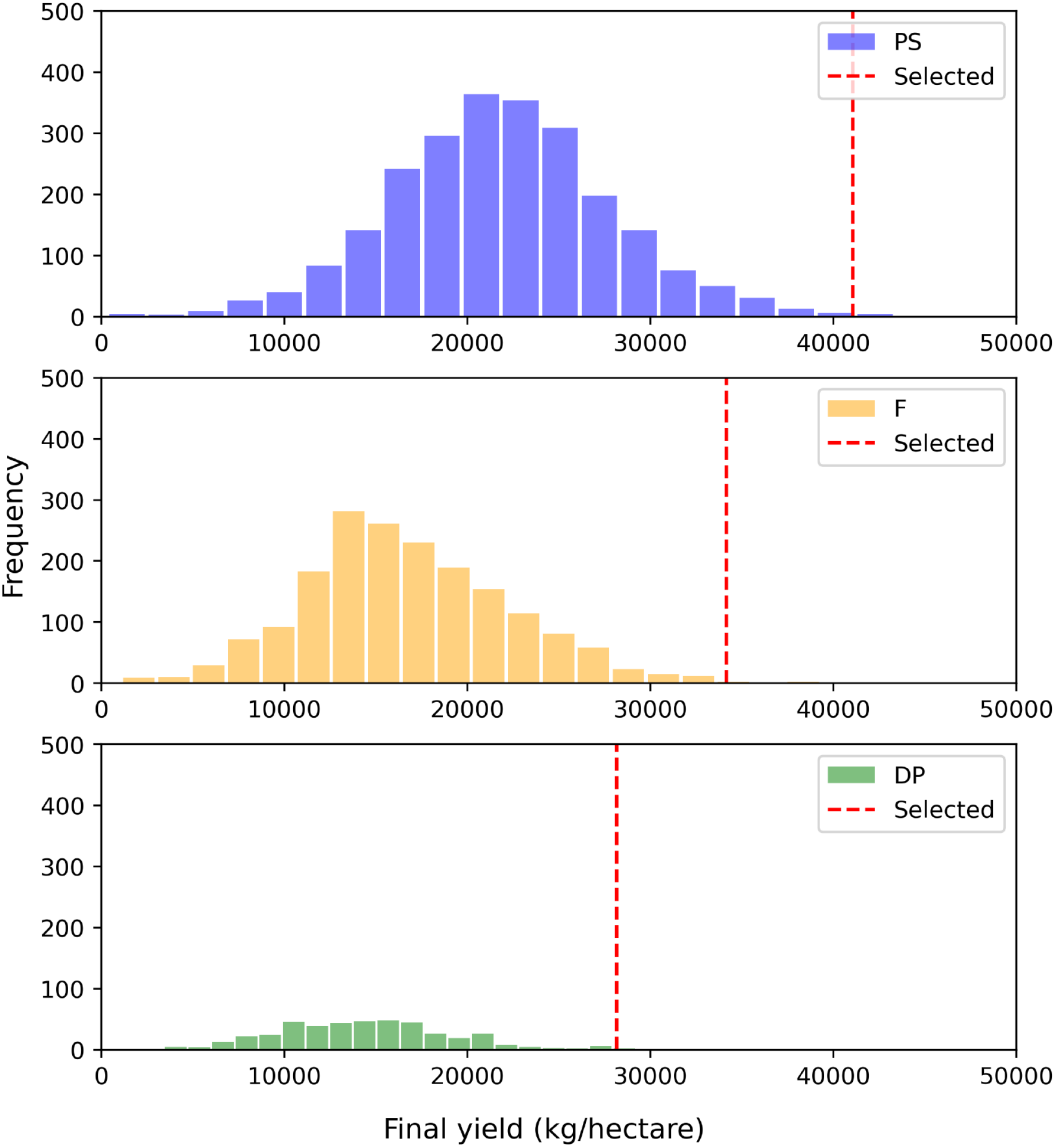
The distribution of true final yield for each plant type and the selected elite individuals from the test set.

**Figure 9** shows the location of the selected elites among the distribution of four plant types. The histogram is based on the original field data in different plant types, while the dashed lines is the selected elite hybrid from the test set. We can see that the selected elites in PS, F, DP all locate at the right tail of the distribution, which shows the effectiveness of our proposed model in distinguishing the highest performers from the general population.

**Figure 10:**
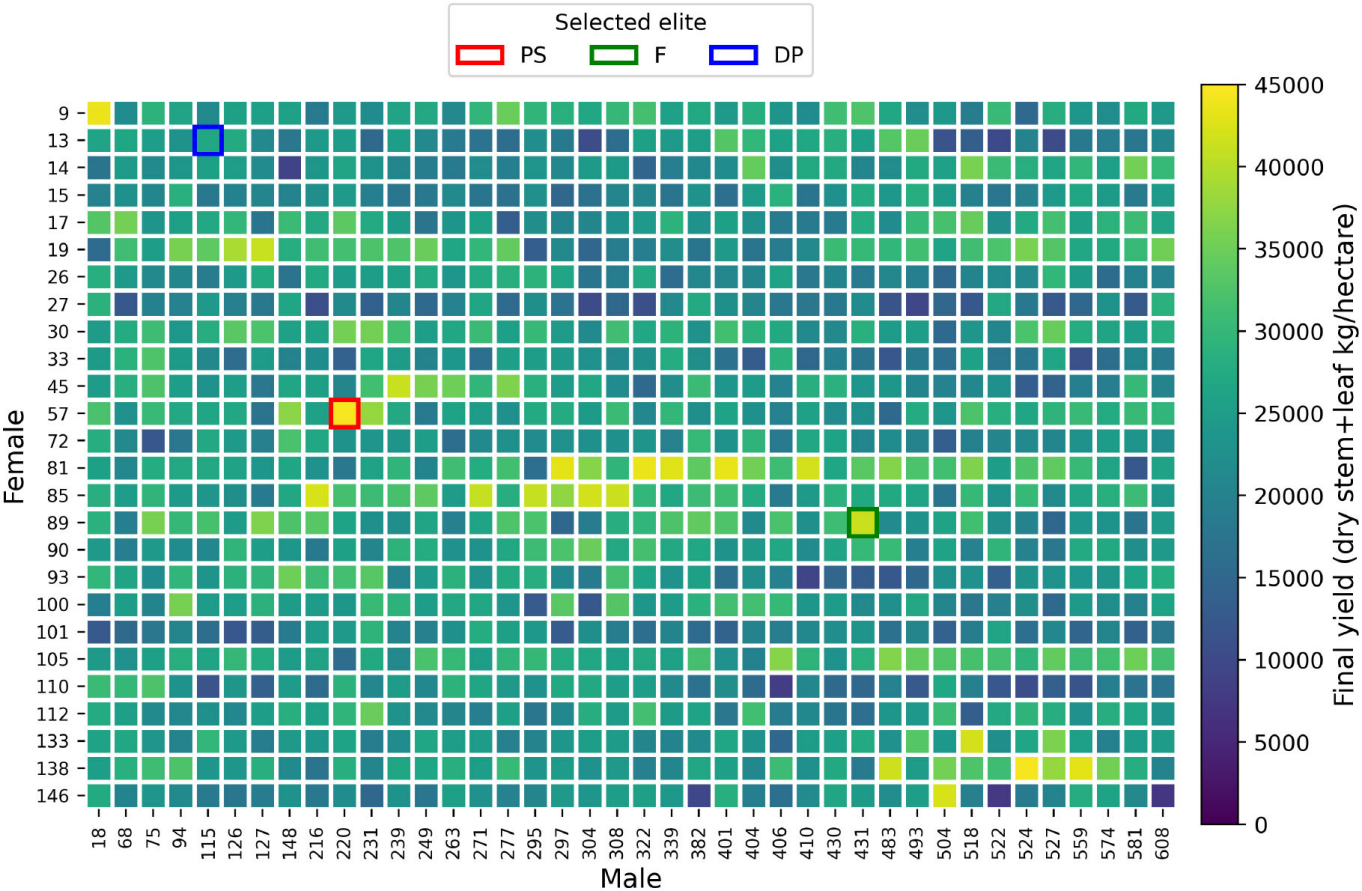
Predicted final yield by parental combination for selected subsets with elite individuals highlighted.

**Figure 10** visualizes the predicted final yield for various combinations of sorghum parents. Each cell in the grid represents a specific male-female pair, and the color intensity reflects the final yield. Here, lighter colors suggest higher yields. As seen in the plots, PS and F elite hybrids perform well in stem development and overall biomass. We can easily distinguish the elite hybrid that we expected based on the heatmap. These visualizations offer a clear view of the genetic interactions driving biomass and grain yield, making it easier for us to identify and select the most promising hybrids.

### 3.3 Genotype by environment interaction in sorghum hybrids

**Figure 11:**
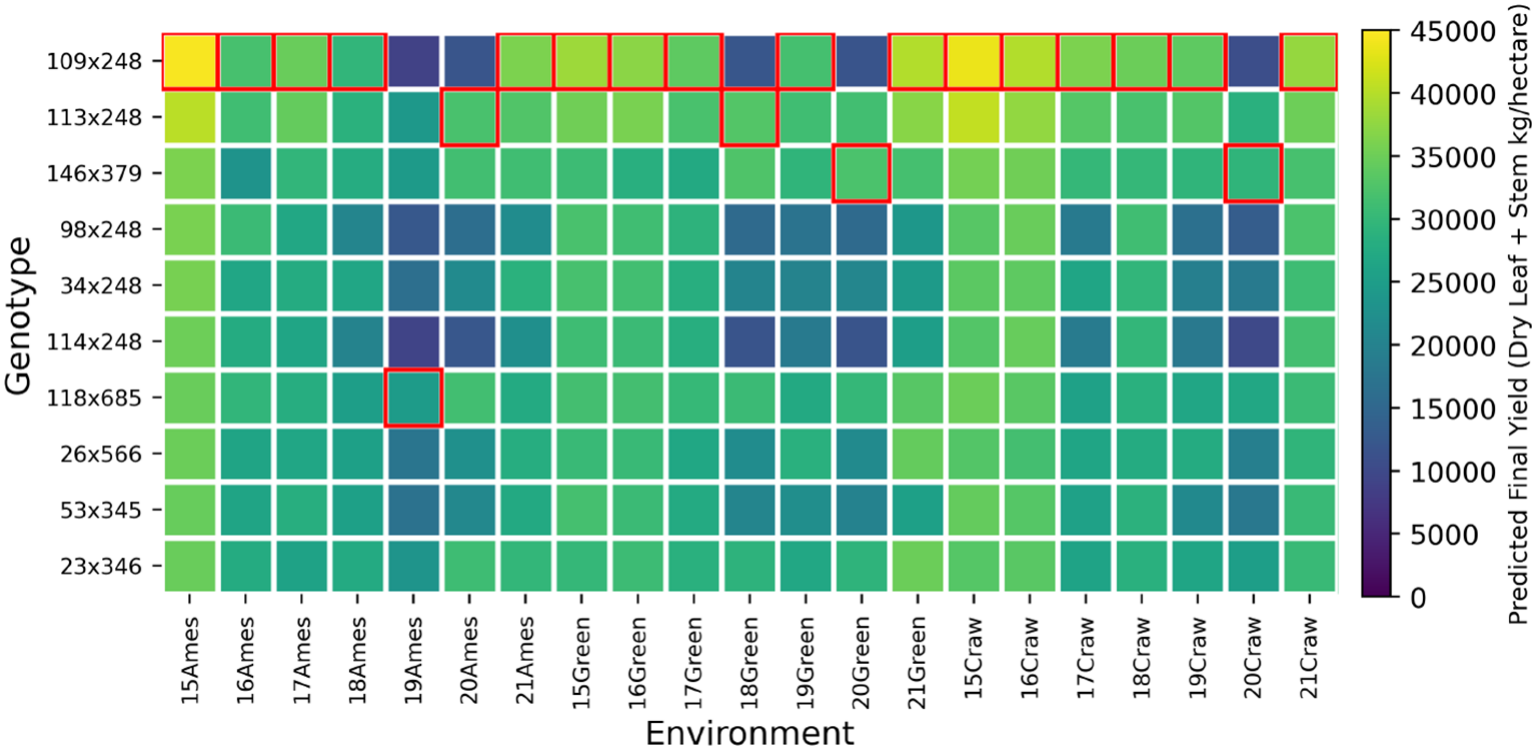
Genotype-by-environment interaction analysis for selected top ten PS Sorghum hybrids.

**Figure 12:**
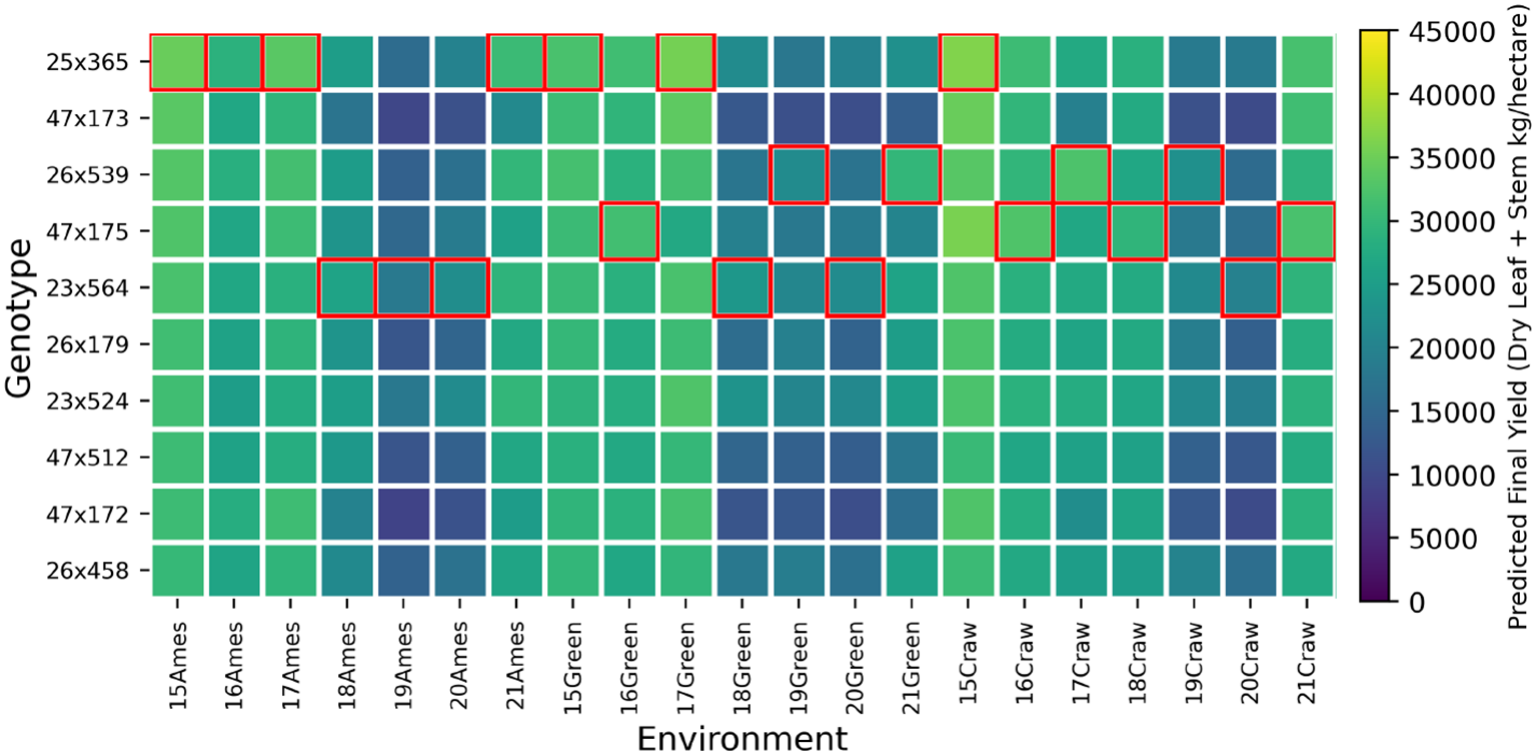
Genotype-by-environment interaction analysis for selected top ten F Sorghum hybrids.

**Figure 13:**
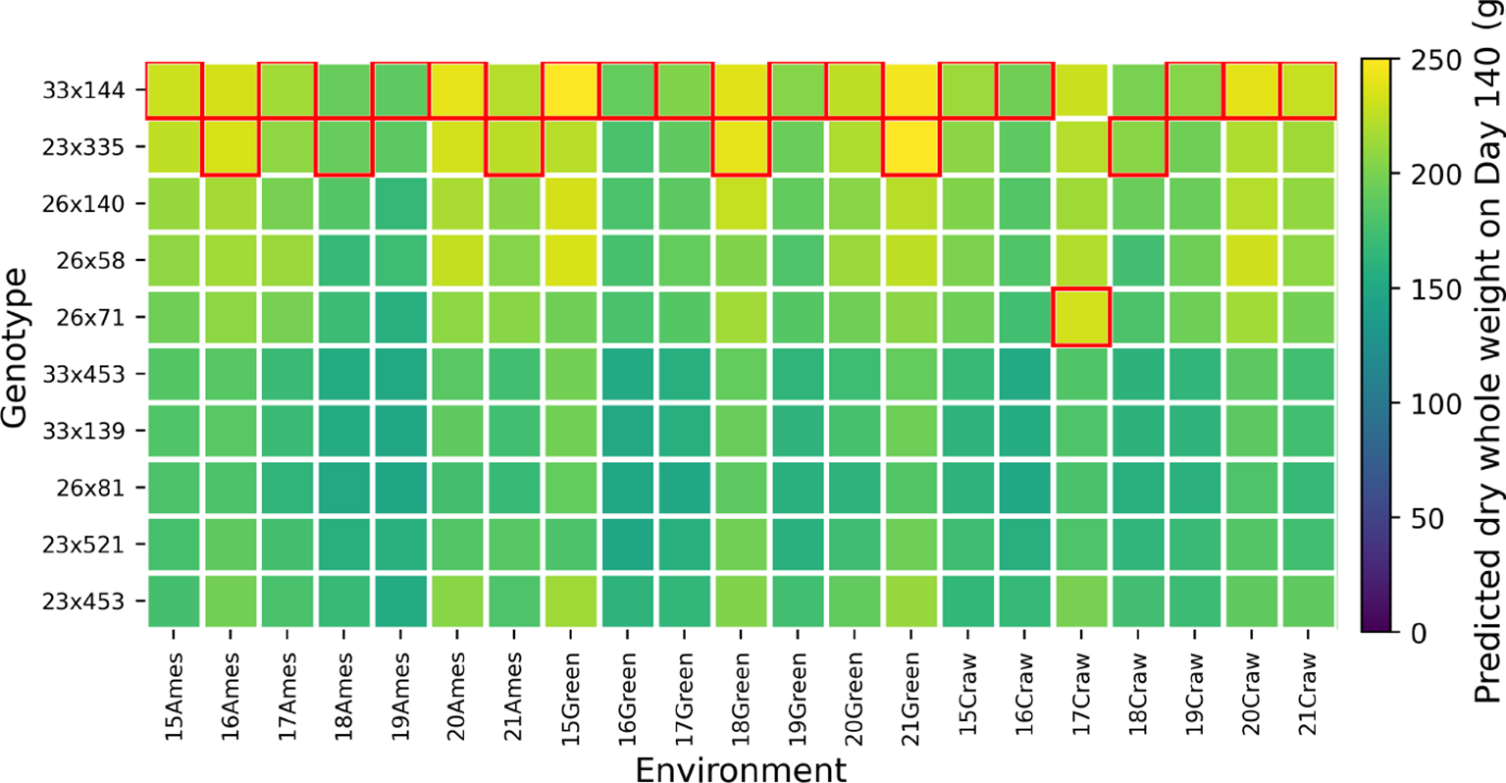
Genotype-by-environment interaction analysis for selected top ten DP Sorghum hybrids.

**Figure 14:**
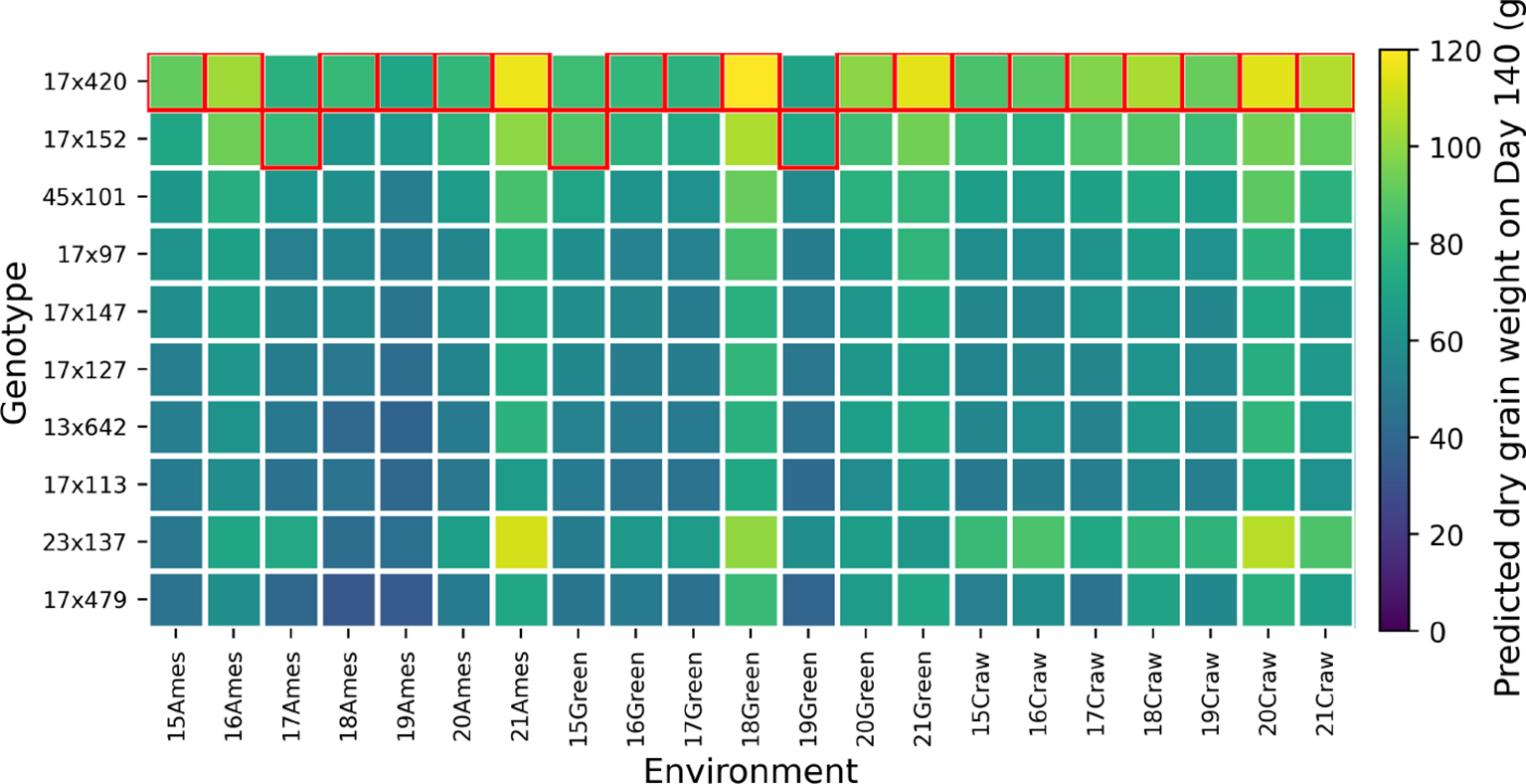
Genotype-by-environment interaction analysis for selected top ten G Sorghum hybrids.

In this section, we explore the genotype by environment (GxE) interaction in the sorghum hybrids based on our proposed model, as illustrated in **Figures 11**–**14**. These figures highlight the significant variability in performance of sorghum elite hybrids (Male x Female) when exposed to different environmental conditions. All 10 hybrids in each plot are elite candidates selected by our model based on the environmental condition in Ames in 2015, ranked from highest to lowest. Based on the weather records, storms occurred in Greenfield in 2018, Ames in 2019, and throughout most Iowa areas in 2020. As a result, significant lodging was observed in these environmental settings. Based on this prior information, we can clearly compare the performance of the hybrids under extreme wind pressure.

**Figure 11** reveals the predicted yield performances of the selected elite PS sorghum individuals. It’s evident that the selected hybrid, 109x248, exhibits outstanding adaptability and yield potential, which are essential traits for coping with rapidly changing climates. However, it suffered from heavy lodging, indicating poor resistance to wind pressure, which is also consistent with our existed records. **Figure 12** shows the predicted yield performances for the selected F sorghum individuals. Here, due to the same selection criterion with PS, we picked the top individuals from the F individuals provided in the test set. From the plot, we can see that the variability of the sorghum performance is comparable larger than the PS type. While the forage types are picked among the existed field records, the phenotypes are more unstable compared to the PS, which also imply the advantage of our model capability.

**Figure 13** shows the predicted whole biomass weight (leaf + stem + grain) for DP type and **Figure 14** represents the predicted dry grain yield on Day 140 for the G type. Different from the PS and F type, these two plant types consider the grain weights, which is sensitive to the environment factors. That result in that these two types have different elite individuals in different environments, which also implies that a decision based on the specific environment condition is vital and essential.

## 4 Conclusion and discussion

Our study developed a bi-stage hybrid model combining explainable neural network and crop modeling to enhance sorghum breeding. The proposed model predicts phenotypic outcomes based on GxE interactions, which revealed strong predictive capability by achieving a cRRMSE of 16% to 19%. The model also successfully detected the elite hybrids for different sorghum types, Grain, Forage, Dual Purpose, and Photoperiod-Sensitive. Our model considers environment, genotype, and management factors and reduces the need for labor-intensive field trials, which is valuable for plant breeders to accelerate the hybrid selection process.

By combining data-driven and process-based models, our proposed bi-stage model not only has a great predictive capability but also has a great interpretability. In the first stage, the model simulates how genotype is translated into intermediate traits known as “trenotypes”, which summarizes the information from the genotype data by incorporating additive, dominant, and epistatic effects. The generated environmentally-independent trenotypes will then be fed to the second stage. This stage will consider the GxE effects to mimic the true growth curve of sorghum based on the provided environment and management condition. A crop physiology model is employed to simulate daily growth activities, such as photosynthesis, transpiration and respiration.

Compared to traditional breeding methods, a key point of our proposed model is that it reduces the necessity of the genotype data by simulating genotype patterns. Genotyping large populations can be expensive and time-consuming, especially in large-scale breeding programs. Our model simulated parental genotype patterns based on the process-based model structure and still maintains predictive power. This makes the model particularly outperformed in resource-limited scenarios. With help of the proposed model, breeders are capable of exploring a wider range of potential hybrids without worrying about the high cost of extensive genotyping process.

However, several areas could further enhance the utility of our proposed model. Currently, our study focuses on homozygous parents and F1 hybrids, without considering recombination events during meiosis. This limitation confines our analysis to a single generation. Expanding the model to include heterozygous parents and multiple generations would allow us to predict more general breeding programs. This will also provide a comprehensive understanding of genetic inheritance patterns and the long-term impact of breeding strategies. Meanwhile, enabling the analysis of recombination events is also critical for understanding the genetic diversity and evolutionary potential of breeding populations.

Furthermore, our experiments are focused on Iowa. Though the time range is large and, thus, encompass a wide variation in weather parameters, the geographic locations still share similar environment patterns. Expanding the dataset to include more diverse environmental conditions and management practices could improve the model’s generalizability. While our model has shown great promise in predicting sorghum growth, its application to other crops remains to be explored. Adapting the model to different species could unlock new opportunities for improving agricultural productivity across a wide range of crops.

In conclusion, our bi-stage hybrid represents a significant step forward in crop breeding technology. By providing accurate and efficient predictions of phenotypes, it supports the discovery of resilient, high-yielding sorghum varieties. Applying the hybrid model to real-world breeding programs and conducting validation trials on newly predicted hybrids will be crucial for further verifying its utility and improving its practical impact on agricultural productivity and sustainability. These efforts will contribute to more resilient and efficient breeding strategies in the face of global agricultural challenges.

## Data Availability

Publicly available datasets were analyzed in this study. This data can be found here: https://mesonet.agron.iastate.edu/agclimate/hist/hourly.php. The codes will be available at: https://github.com/TroubleZN/Bi-stage-data-driven-process-based-model-for-sorghum-breeding-and-yield-prediction}{Github}.'?
