# Supplementary figures and images for: A bi-stage data-driven process-based model for sorghum breeding and yield prediction: coupling explainable artificial intelligence and crop modeling
Source: Front Plant Sci. 2026 Jan 8;16:1617753. doi: 10.3389/fpls.2025.1617753 (PMC12823968; doi:10.3389/fpls.2025.1617753)

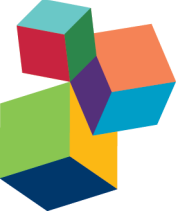

Supplement: Supplementary file 1 [file SupplementaryFile1.zip › _Accepted__A_bi_stage_data_driven_process_based_model_for_sorghum_breeding_and_yield_prediction/logo2.pdf]

A

frontiers  
FOR YOUNG MINDS

B

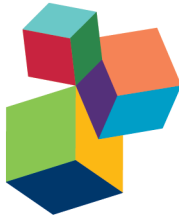

Supplement: Supplementary file 1 [file SupplementaryFile1.zip › _Accepted__A_bi_stage_data_driven_process_based_model_for_sorghum_breeding_and_yield_prediction/logos.pdf]

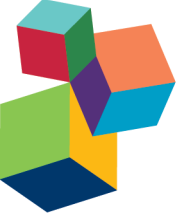

frontiers

Supplement: Supplementary file 1 [file SupplementaryFile1.zip › _Accepted__A_bi_stage_data_driven_process_based_model_for_sorghum_breeding_and_yield_prediction/logo1.pdf]

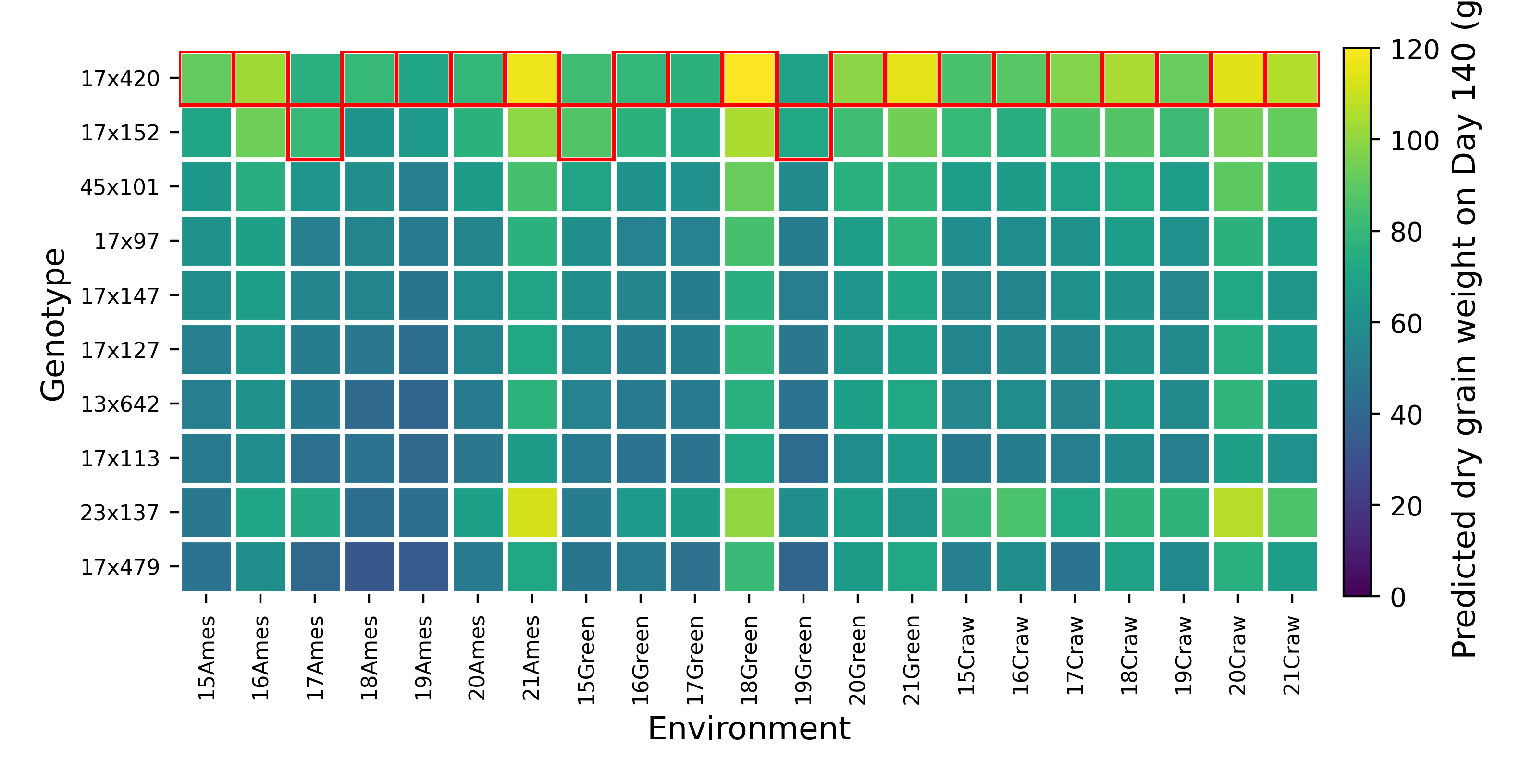

Supplement: Supplementary file 1 [file SupplementaryFile1.zip › _Accepted__A_bi_stage_data_driven_process_based_model_for_sorghum_breeding_and_yield_prediction/Fig/GxE_G.png]

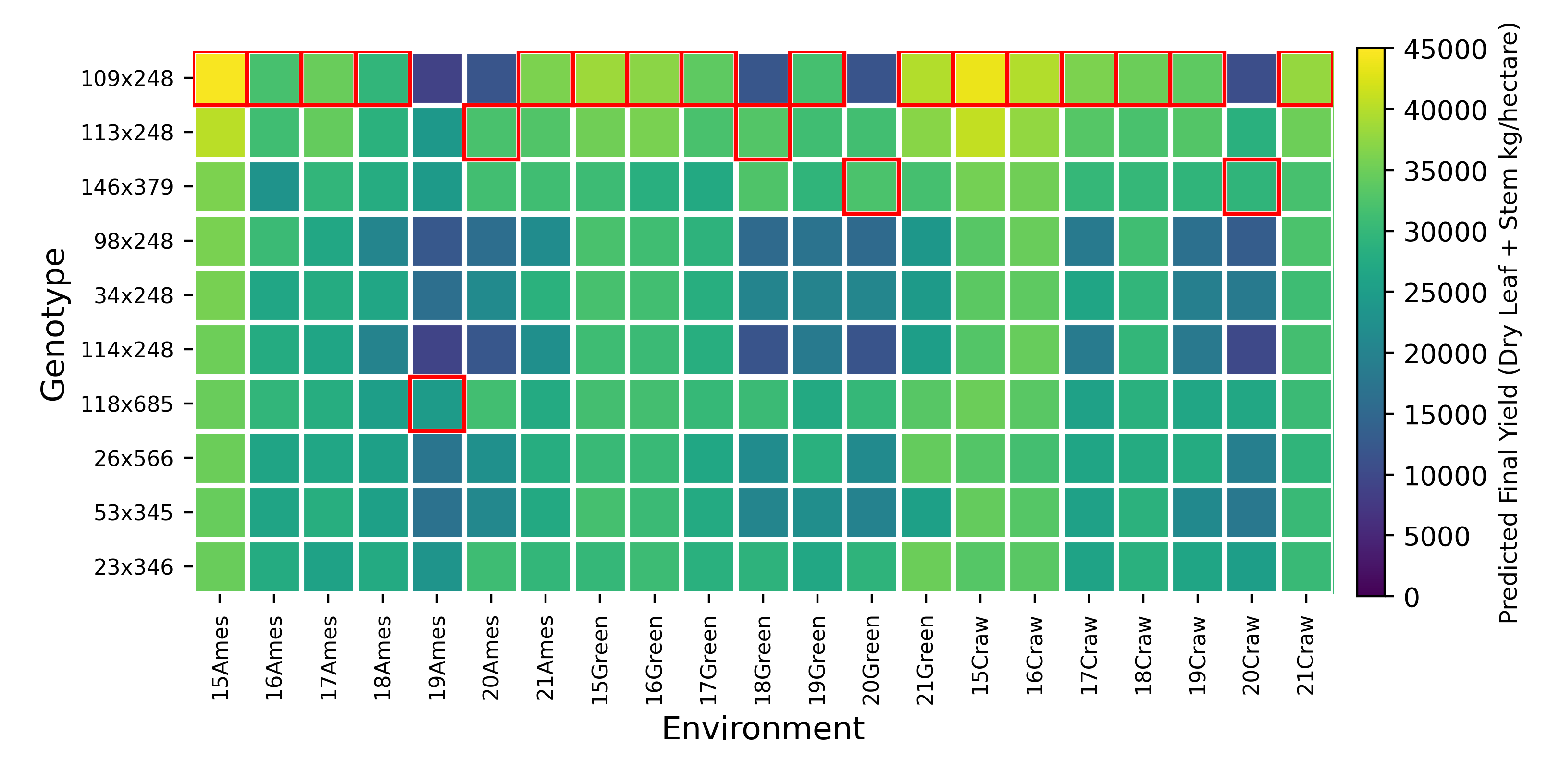

Supplement: Supplementary file 1 [file SupplementaryFile1.zip › _Accepted__A_bi_stage_data_driven_process_based_model_for_sorghum_breeding_and_yield_prediction/Fig/GxE_PS.png]

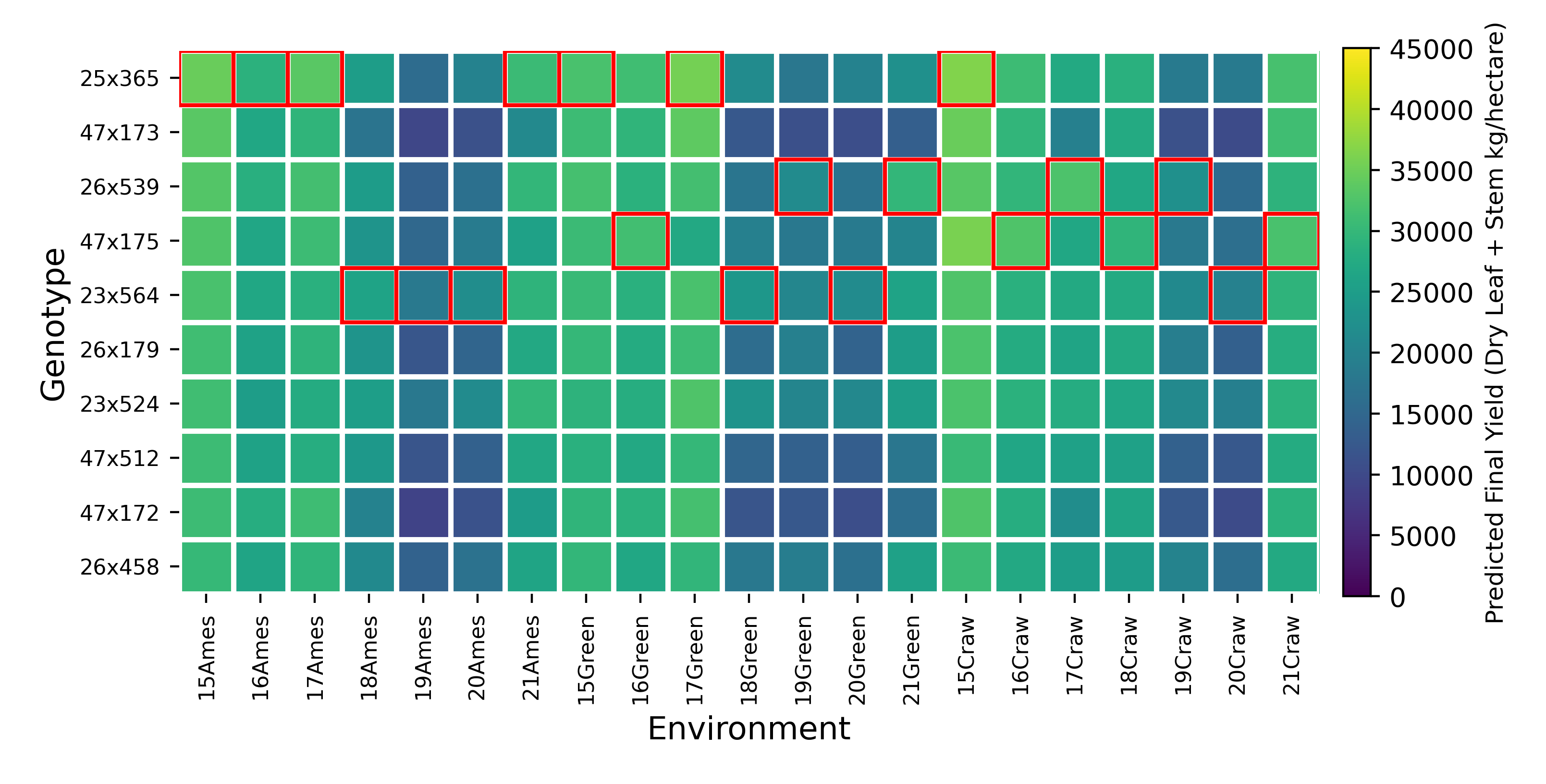

Supplement: Supplementary file 1 [file SupplementaryFile1.zip › _Accepted__A_bi_stage_data_driven_process_based_model_for_sorghum_breeding_and_yield_prediction/Fig/GxE_F.png]

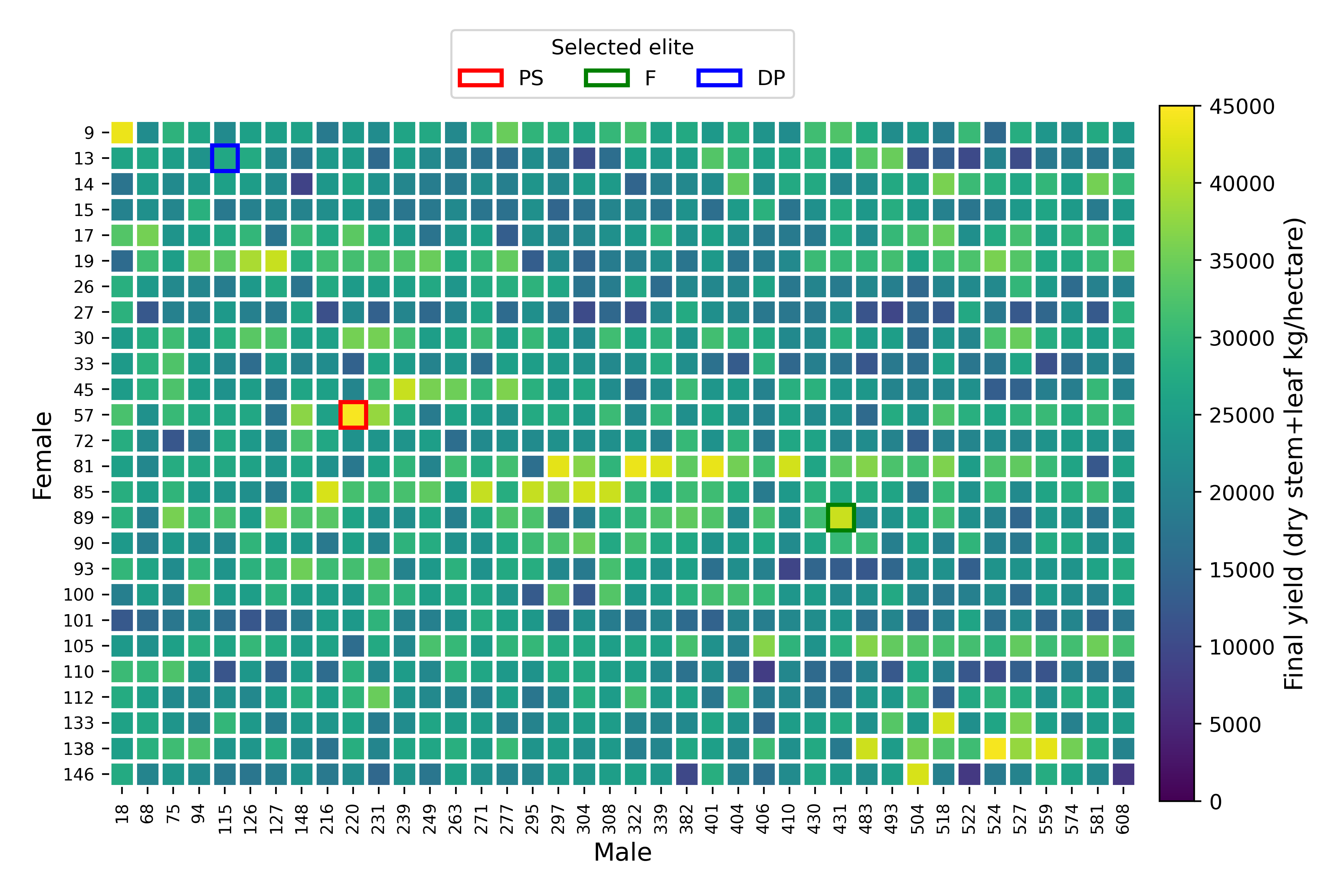

Supplement: Supplementary file 1 [file SupplementaryFile1.zip › _Accepted__A_bi_stage_data_driven_process_based_model_for_sorghum_breeding_and_yield_prediction/Fig/Yield_elite.png]

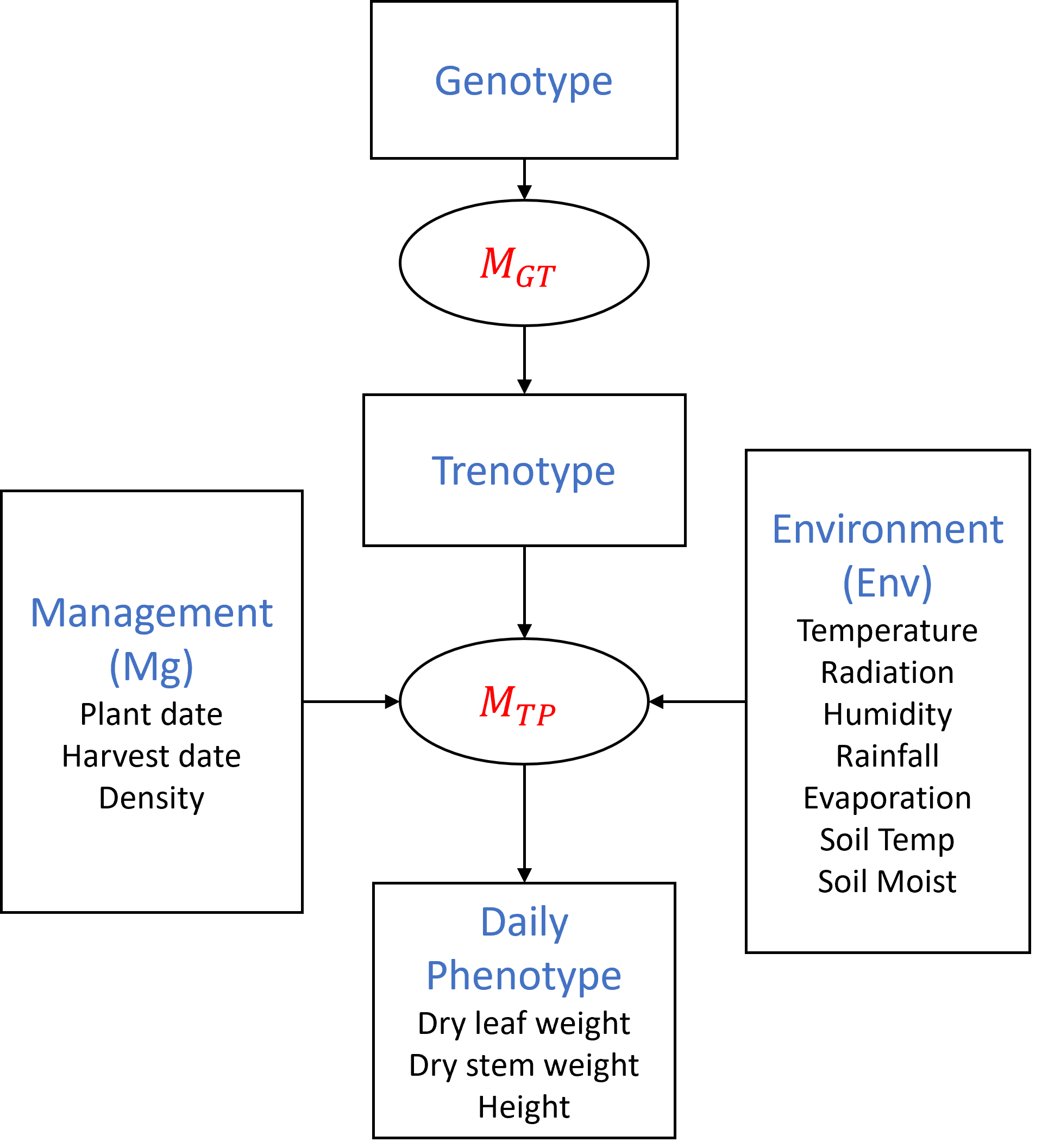

Supplement: Supplementary file 1 [file SupplementaryFile1.zip › _Accepted__A_bi_stage_data_driven_process_based_model_for_sorghum_breeding_and_yield_prediction/Fig/General_structure.png]

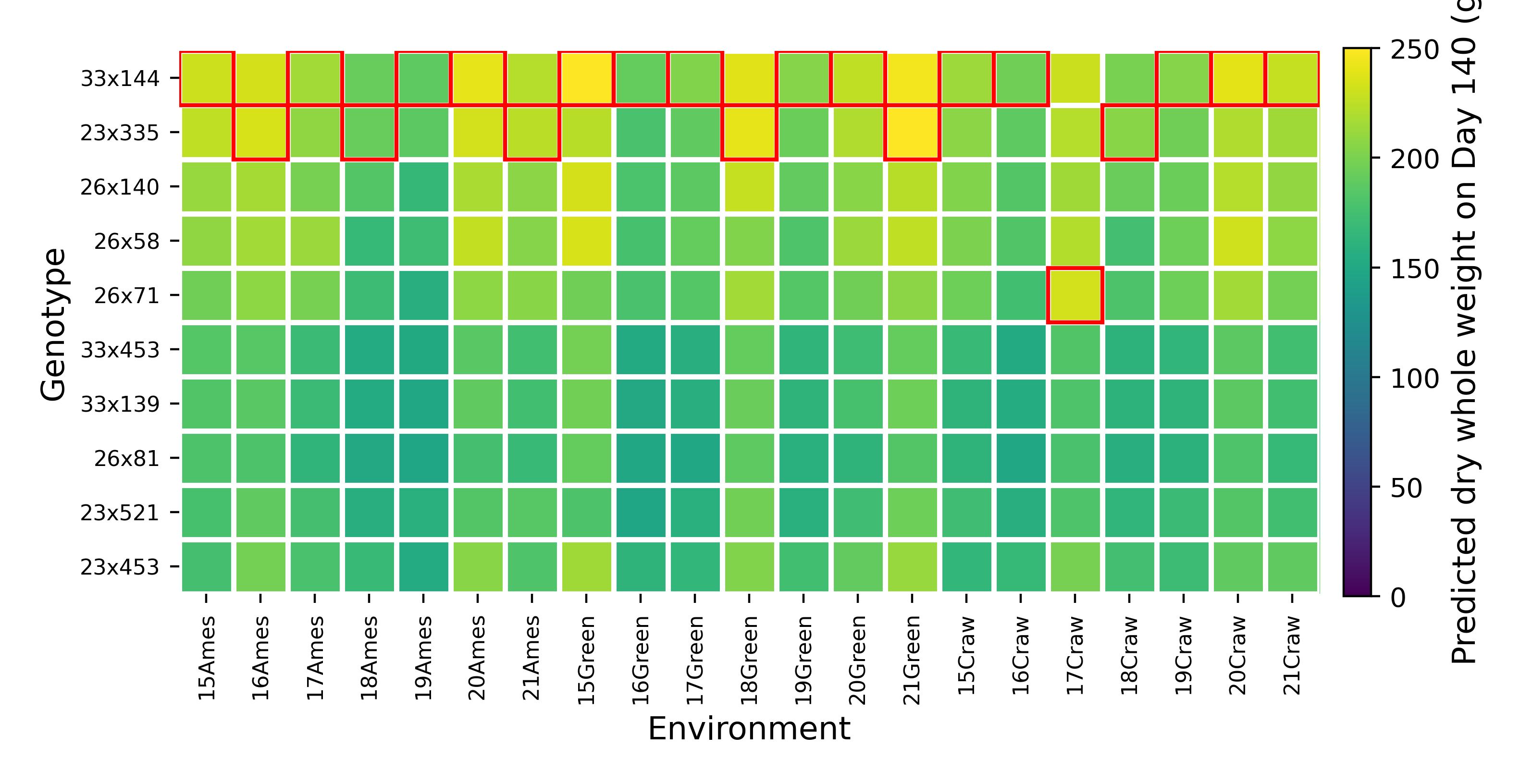

Supplement: Supplementary file 1 [file SupplementaryFile1.zip › _Accepted__A_bi_stage_data_driven_process_based_model_for_sorghum_breeding_and_yield_prediction/Fig/GxE_DP.png]

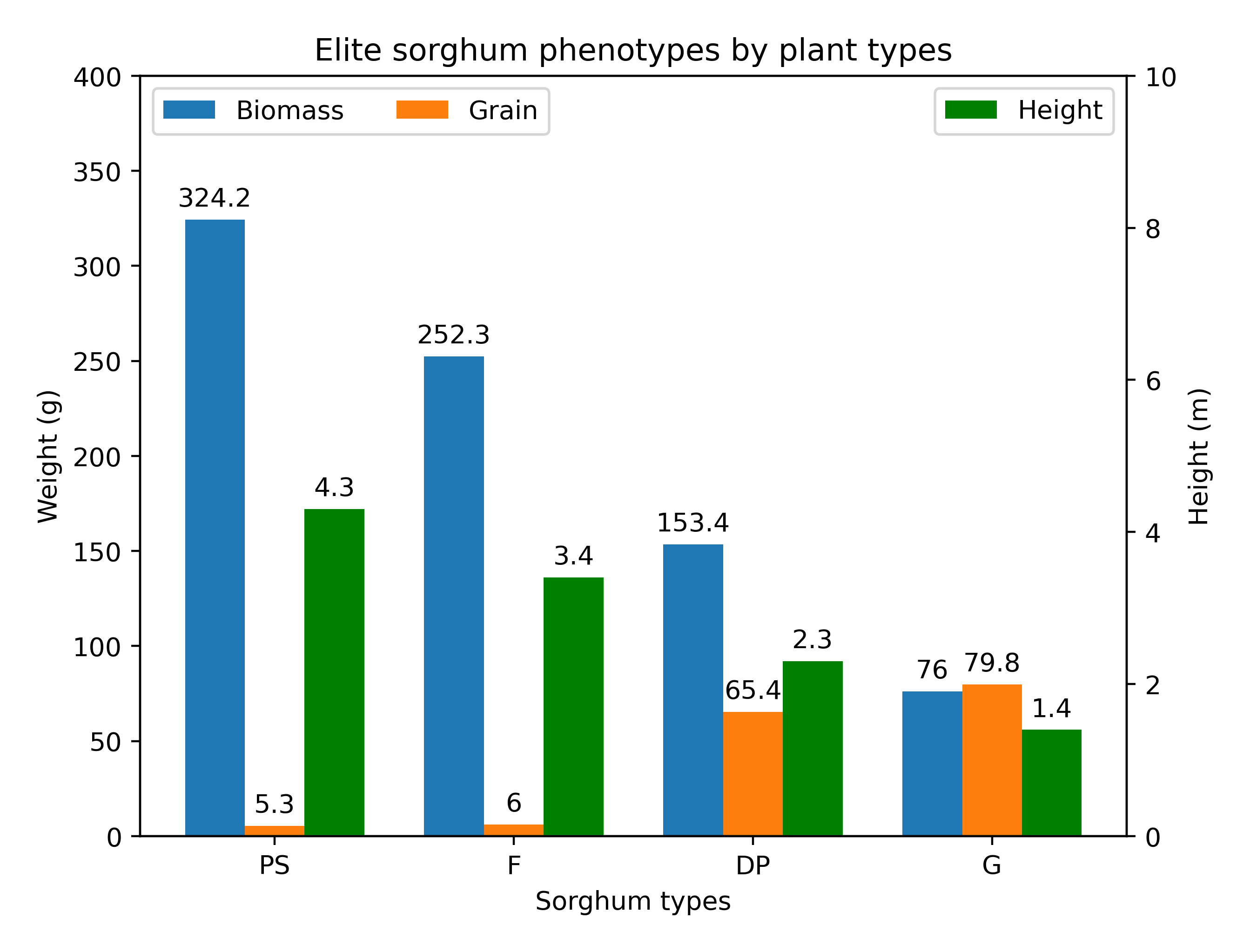

Supplement: Supplementary file 1 [file SupplementaryFile1.zip › _Accepted__A_bi_stage_data_driven_process_based_model_for_sorghum_breeding_and_yield_prediction/Fig/elite_phenotype.png]

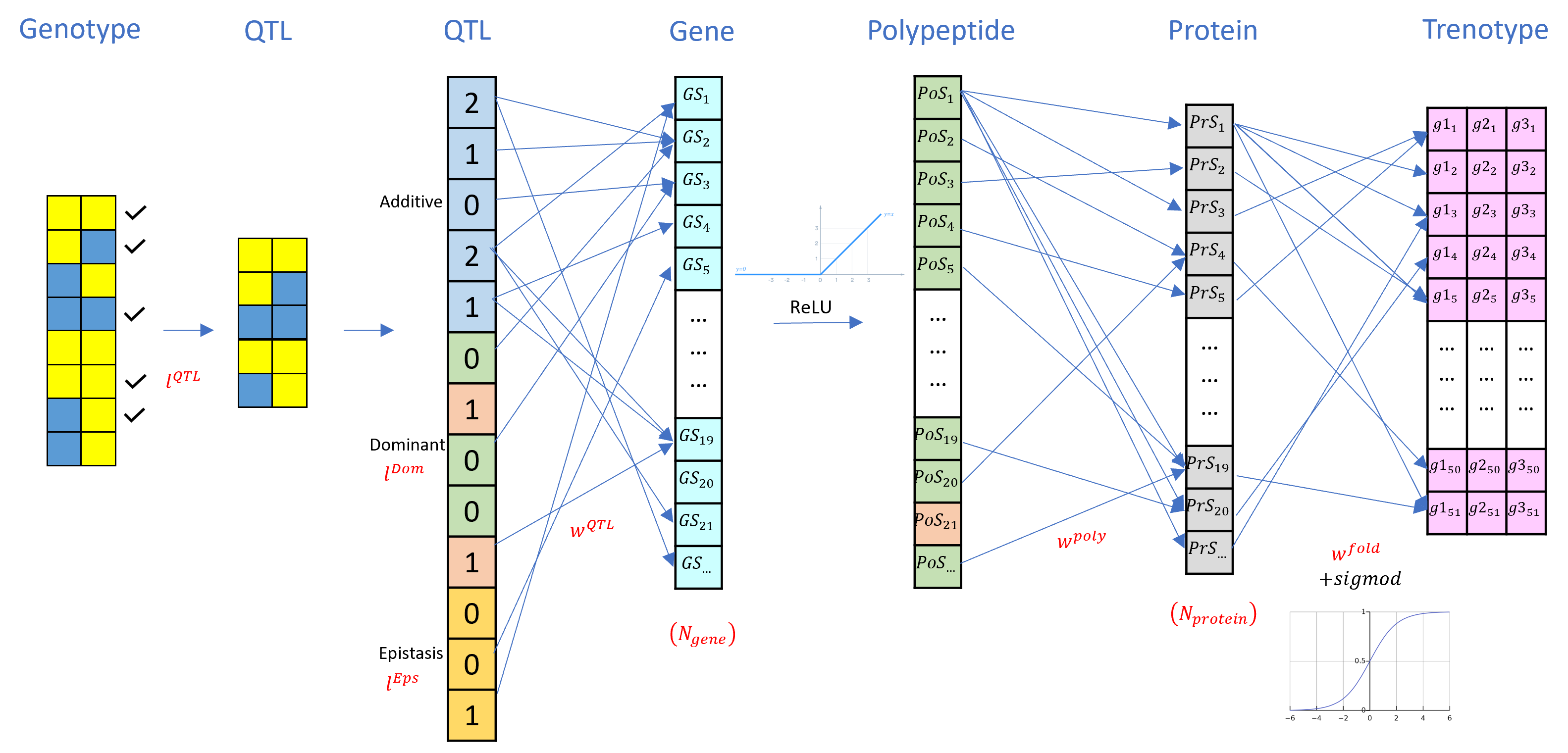

Supplement: Supplementary file 1 [file SupplementaryFile1.zip › _Accepted__A_bi_stage_data_driven_process_based_model_for_sorghum_breeding_and_yield_prediction/Fig/M_GT.png]

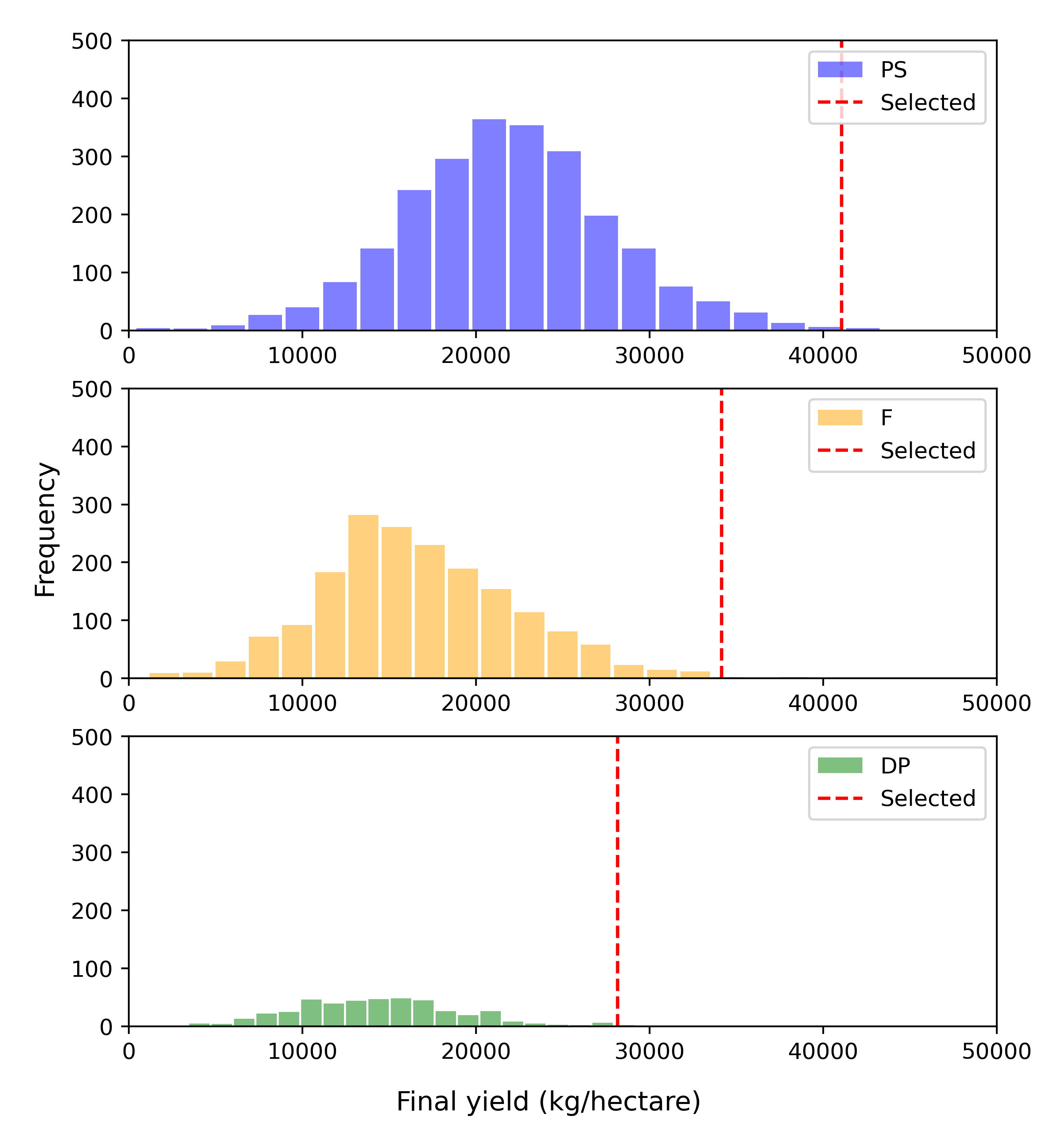

Supplement: Supplementary file 1 [file SupplementaryFile1.zip › _Accepted__A_bi_stage_data_driven_process_based_model_for_sorghum_breeding_and_yield_prediction/Fig/Yield_distribution.png]

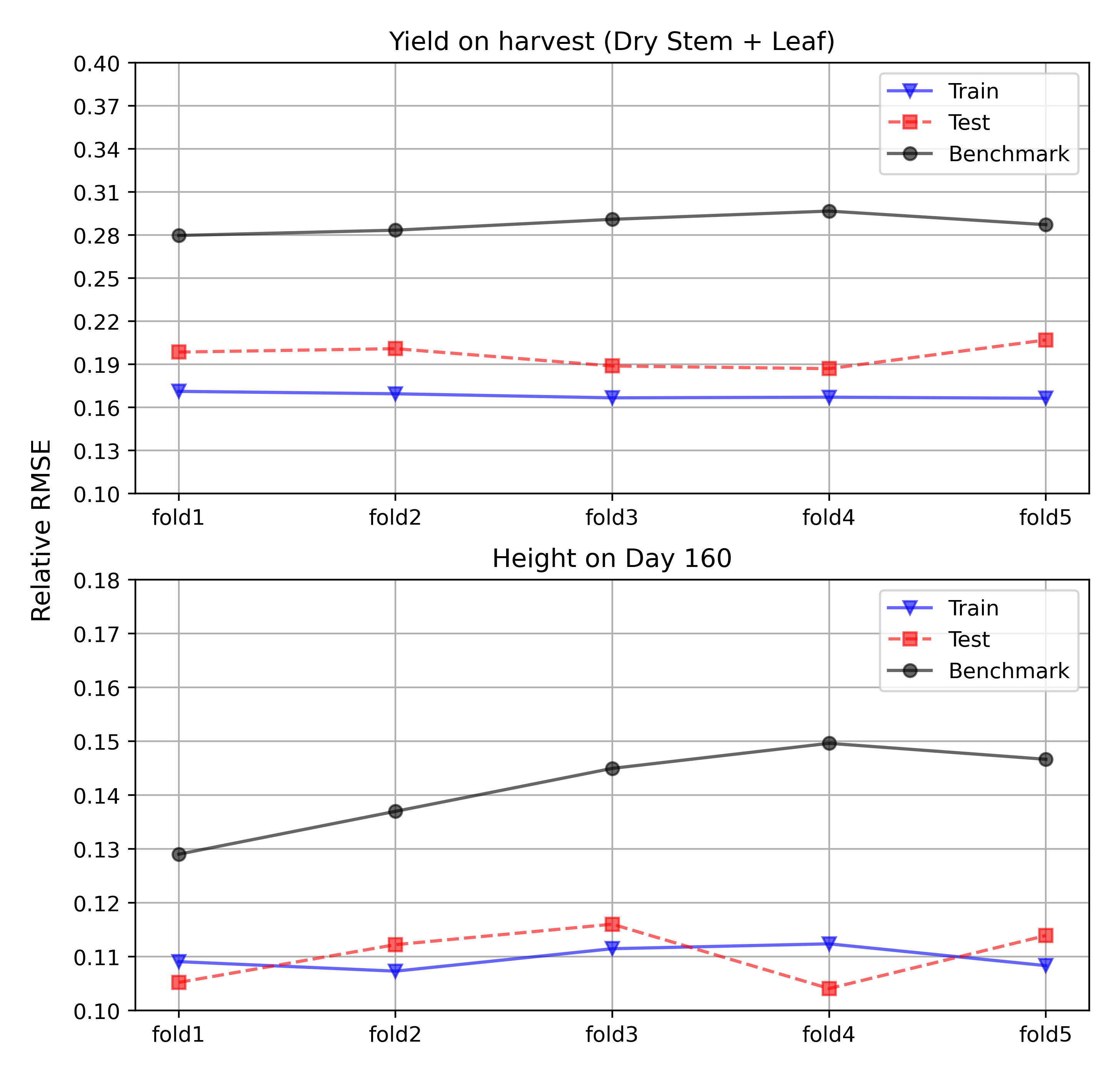

Supplement: Supplementary file 1 [file SupplementaryFile1.zip › _Accepted__A_bi_stage_data_driven_process_based_model_for_sorghum_breeding_and_yield_prediction/Fig/RRMSE.png]

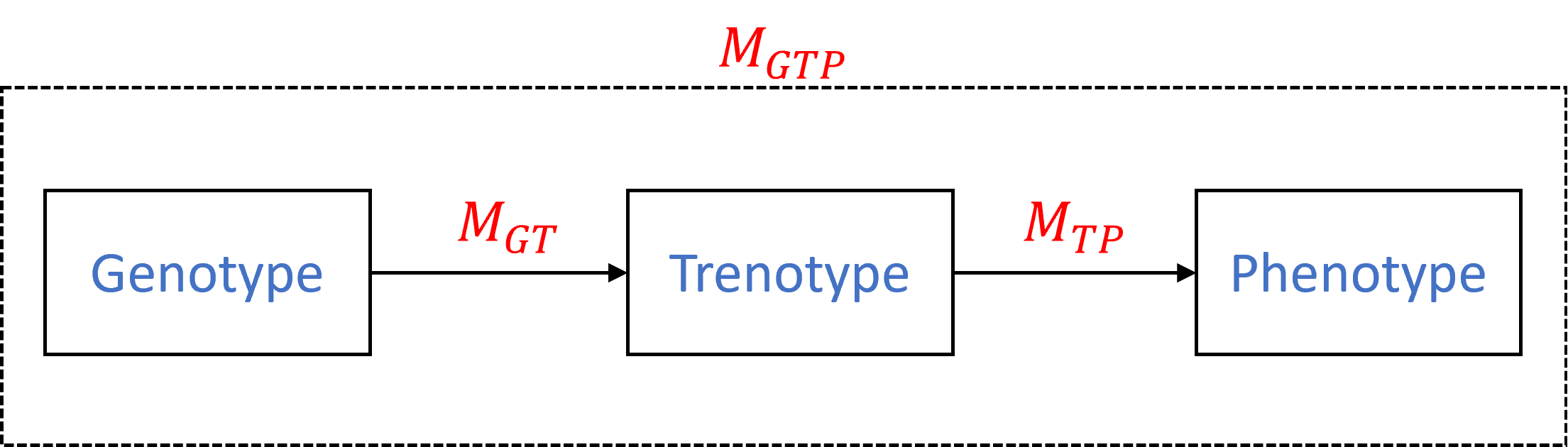

Supplement: Supplementary file 1 [file SupplementaryFile1.zip › _Accepted__A_bi_stage_data_driven_process_based_model_for_sorghum_breeding_and_yield_prediction/Fig/Model_annotations.png]

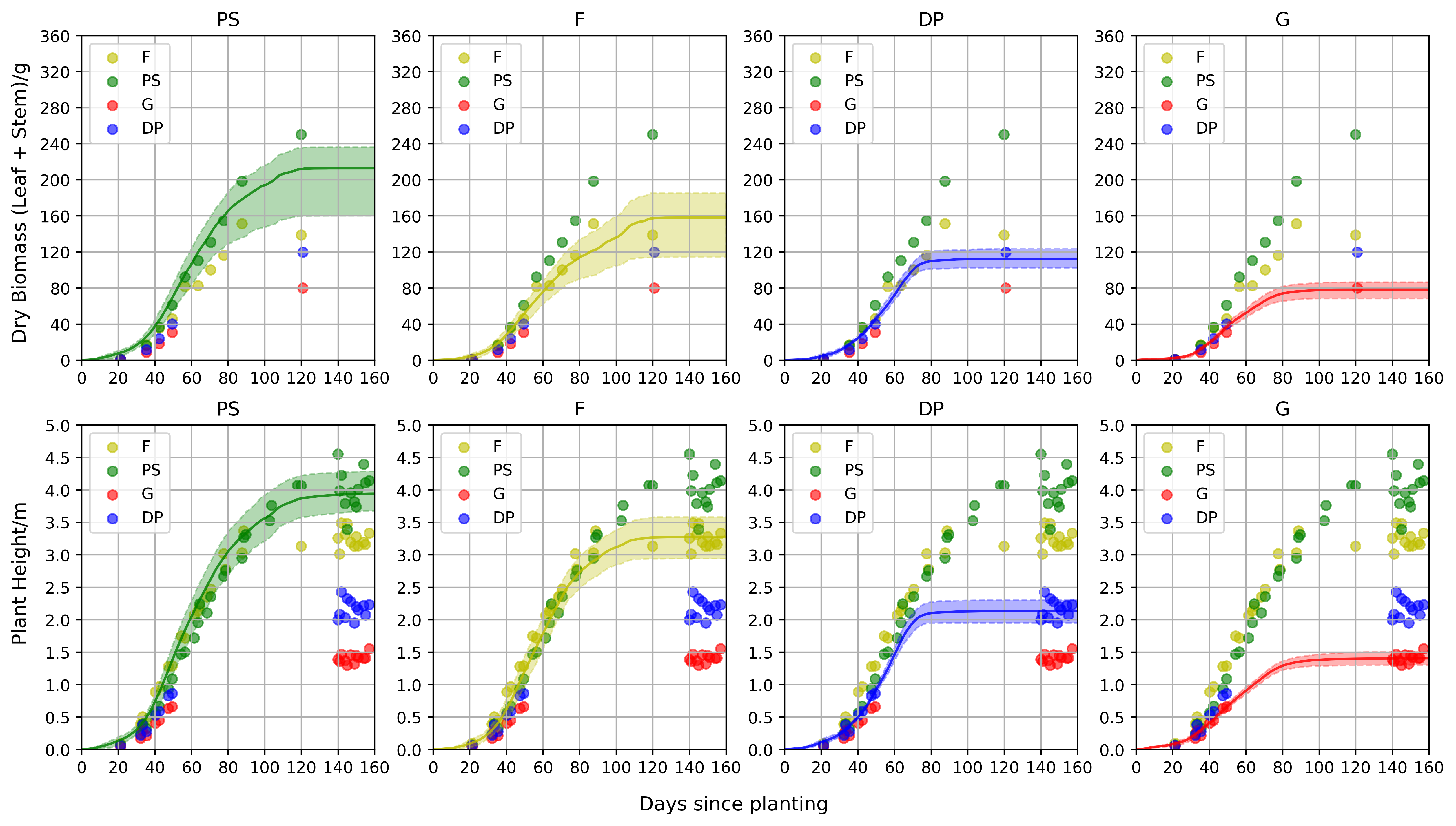

Supplement: Supplementary file 1 [file SupplementaryFile1.zip › _Accepted__A_bi_stage_data_driven_process_based_model_for_sorghum_breeding_and_yield_prediction/Fig/Predictive_Capability.png]

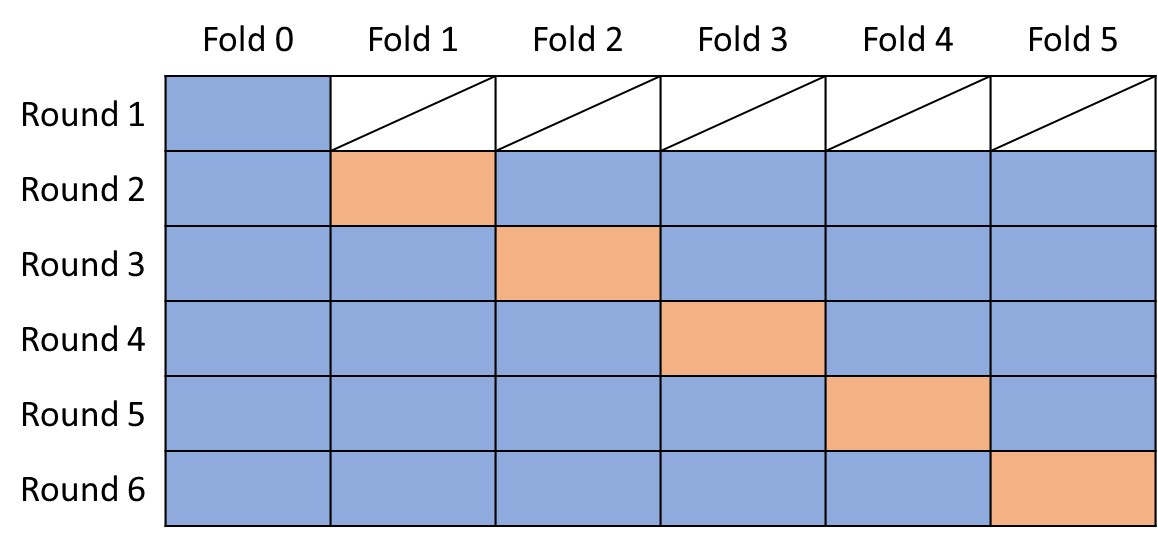

Supplement: Supplementary file 1 [file SupplementaryFile1.zip › _Accepted__A_bi_stage_data_driven_process_based_model_for_sorghum_breeding_and_yield_prediction/Fig/CV.jpg]

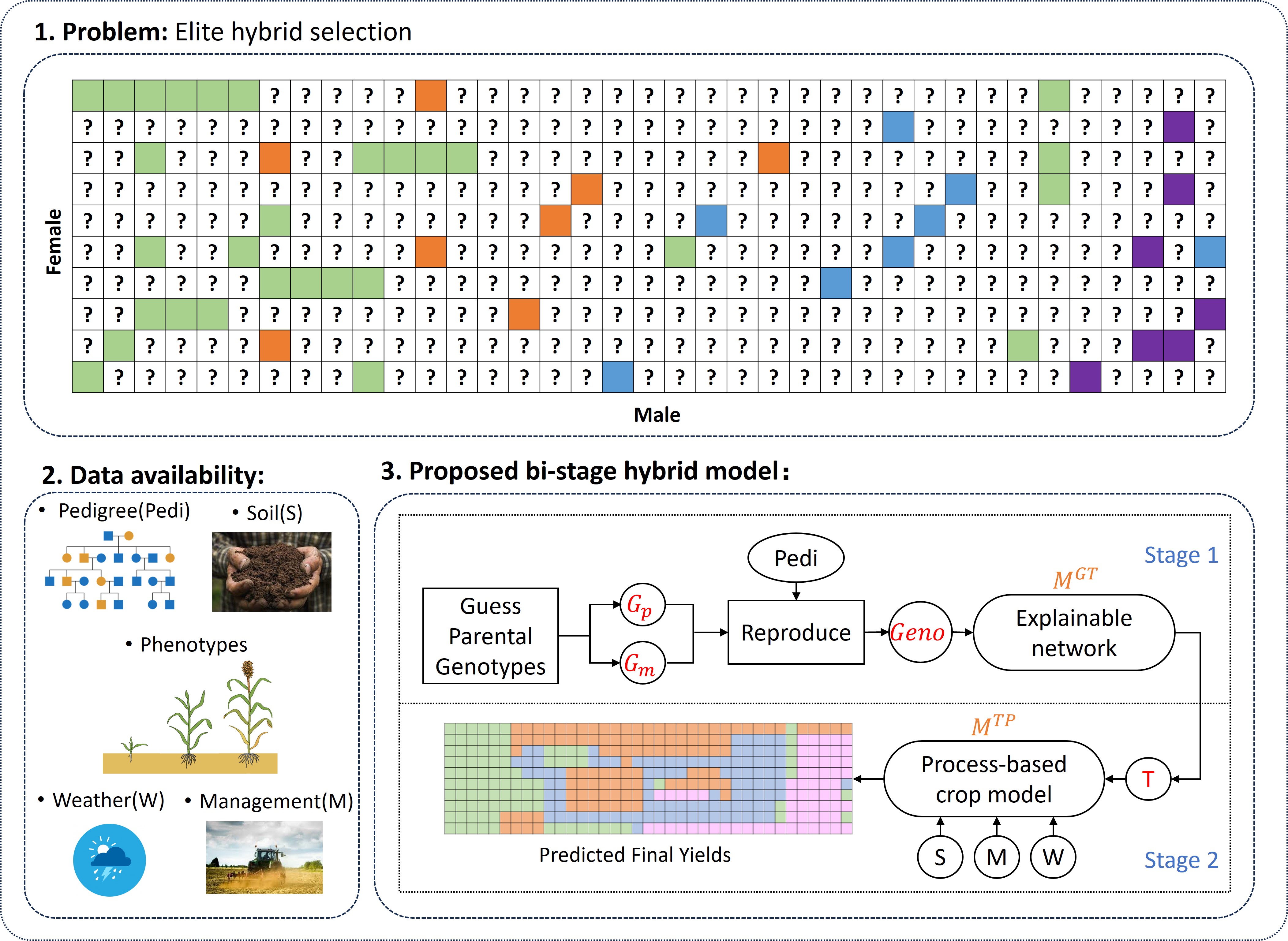

Supplement: Supplementary file 1 [file SupplementaryFile1.zip › _Accepted__A_bi_stage_data_driven_process_based_model_for_sorghum_breeding_and_yield_prediction/Fig/Introduction.jpg]

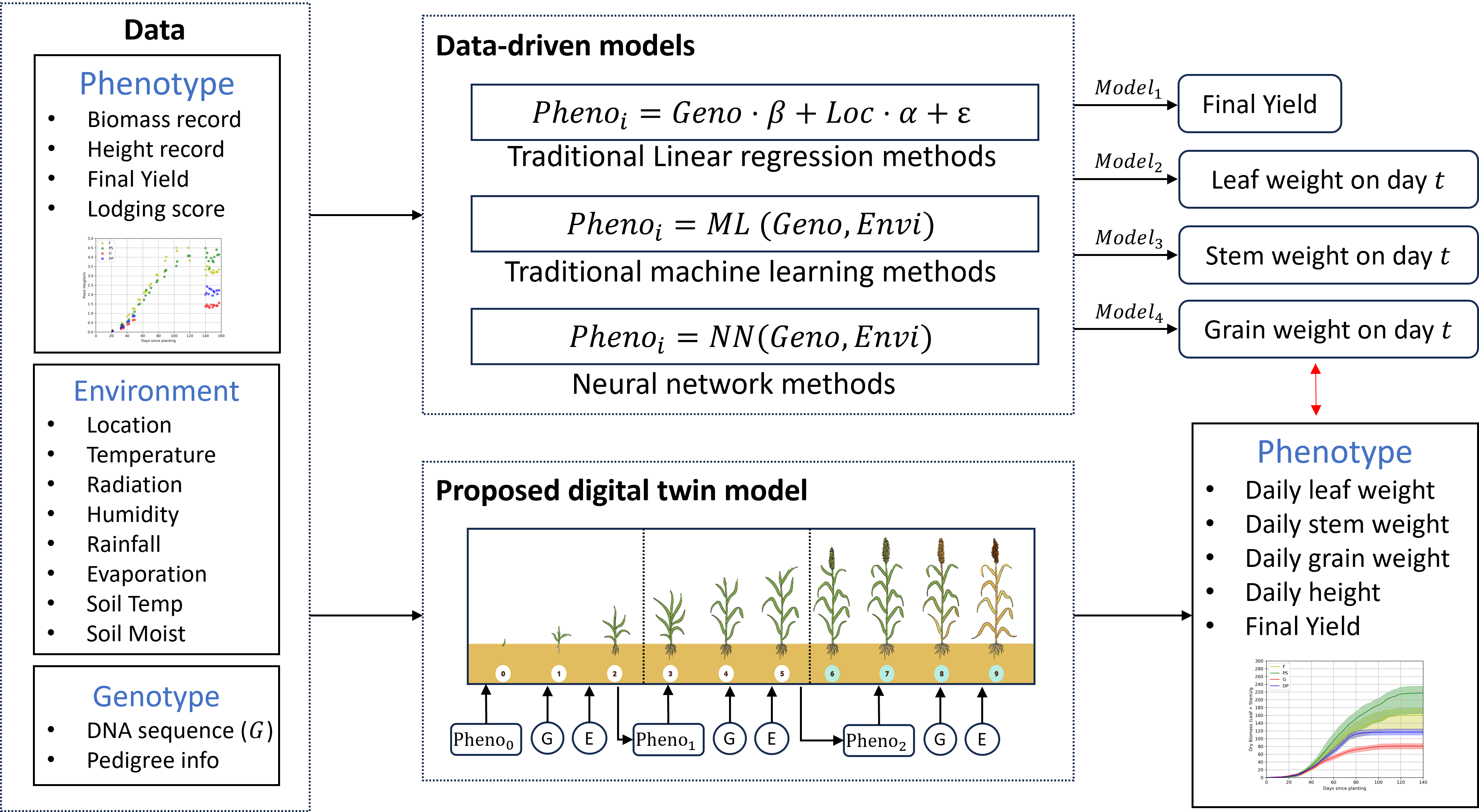

Supplement: Supplementary file 1 [file SupplementaryFile1.zip › _Accepted__A_bi_stage_data_driven_process_based_model_for_sorghum_breeding_and_yield_prediction/Fig/Model_comparison.png]
